# Sedentary Behavior and Health Consequences: A Systematic Scoping Review of Prospective and Longitudinal Studies in Japan

**DOI:** 10.2188/jea.JE20250140

**Published:** 2026-01-05

**Authors:** Ai Shibata, Kaori Ishii, Neville Owen, Koichiro Oka

**Affiliations:** 1 Institute of Health and Sport Sciences, University of Tsukuba, Ibaraki, Japan; 2 Faculty of Sport Sciences, Waseda University, Saitama, Japan; 3 School of Health Sciences, Swinburne University of Technology, Melbourne, Victoria, Australia; 4 Physical Activity Laboratory, Baker Heart & Diabetes Institute, Melbourne, Victoria, Australia

**Keywords:** sitting, Japanese, adults, cohort, health risk

## Abstract

**Objectives:**

This scoping review summarizes and evaluates evidence from Japan on prospective relationships of sedentary behavior (too much sitting, as distinct from too little physical activity) with health outcomes, forming the basis for Japan’s new sedentary behavior guidelines. It also identified evidence gaps and provided recommendations for future public health guidelines.

**Methods:**

A systematic search was conducted in PubMed, Web of Science, CINAHL, and MEDLINE for English-language, peer-reviewed longitudinal studies on sedentary behavior and health outcomes in apparently healthy Japanese adults published between 2000 and 2023. The search strategy was developed based on sedentary behavior measures, study design, and study population. Two independent reviewers screened titles, abstracts, and full texts. Data were synthesized narratively, with a quality assessment performed.

**Results:**

Twenty-seven relevant studies were identified, all but one published after 2013. About half focused on middle-aged and older adults, primarily using self-report questionnaires. Many studies were large cohorts (>10,000 participants) with follow-ups of more than 10 years. Studies varied widely in physical activity indicators, confounders, time classifications, and cutoff values for sedentary behavior. The studies examined 29 health outcomes, primarily all-cause mortality, cancer incidence, and cancer mortality. Most studies reported at least partial evidence of harmful associations between sedentary behavior and health outcomes, though only eight were rated as good quality.

**Conclusion:**

There is sufficient evidence to support minimizing sedentary time to promote health in Japanese adults. However, due to the limited number of high-quality studies, the specificity and dose-response relationship between sedentary behavior and health outcomes remain unclear.

## INTRODUCTION

Sedentary behavior is distinct from lack physical activity and is defined as any waking behavior characterized by an energy expenditure ≤1.5 metabolic equivalents (METs) while in a sitting, reclining, or lying posture.^1^ It is increasingly prevalent in daily life due to technological advancements affecting commuting, workplaces, and leisure activities. Sedentary behavior accounts for 55 to 60% of waking hours on average globally, equating to some 8–9 hours per day.^2^ This trend has been exacerbated by societal shifts, particularly during the COVID-19 pandemic.^3^^,^^4^ Excessive sedentary time is particularly concerning in Japan. A previous international study examining self-reported sitting time across 20 countries with diverse population profiles ranked Japan as the second most sedentary nation, with a median sitting time of 7 hours per day.^5^

Excessive sedentary behavior has emerged as a significant public health issue among adults worldwide. Over the past two decades, research on the health risks associated with sedentary behavior has advanced rapidly and broadly, particularly in Western countries. The clear scientific evidence from systematic reviews and meta-analyses of prospective observational studies shows a high level of sedentary behavior to be related to an increased risk of all-cause, cardiovascular, and cancer mortalities, as well as incidence of cardiovascular diseases (CVDs) and type 2 diabetes, even after adjusting moderate-to vigorous intensity physical activity (MVPA).^6^^–^^8^ Furthermore, these meta-analyses have reported a progressively increasing risk of all-cause mortality with more than approximately 6 to 9.5 hours of sedentary behavior per day.^6^^,^^8^ Given the weak temporal trade-off between sedentary behavior and MVPA,^9^ public health efforts should focus on reducing excessive sedentary behavior alongside promoting regular MVPA participation. Accordingly, in 2020, the World Health Organization (WHO) incorporated the term of “sedentary behavior” into the title of its physical activity guidelines (Physical Activity and Sedentary Behavior) and, for the first time, issued recommendation on reducing sedentary behavior within the guidelines.^10^ However, specific recommendations regarding the thresholds or minimum levels of total sitting time that may cause various health risks, as well as effective and specific patterns for interrupting sedentary behavior, were not included due to insufficient evidence on the dose-response relationship with various health indicators based on a comprehensive systematic review.^11^

The official Japanese physical activity guidelines for health promotion were revised in 2023 for the first time in 10 years. The updated guidelines recommend that Japanese adults aged 18–64 years engage in 60 minutes of MVPA per day without specific consideration of bout duration, which is consistent with the previous guidelines published in 2013.^12^^,^^13^ This recommendation was established based on a rigorous meta-analysis of relevant prospective observational studies. Consequently, the recommended amount of physical activity for Japan is substantially higher than that of the United States and WHO, partly due to differences in the baseline physical activity levels of the population.^13^

Regarding sedentary behavior, a recommendation was newly introduced in the 2023 Japanese physical activity guidelines, aligning with WHO’s guidelines: “Be careful not to sit for too long”.^12^ Before incorporating sedentary behavior recommendations into the public guidelines, it was crucial to determine whether recommendations for reducing sedentary behavior should be included and whether more time- and pattern-specific guidelines should be developed as part of public health guidelines, even if limited to domestic application. Thus, identifying the specificity and dose-response relationship between sedentary behavior and health outcomes is essential.^14^

Building on evidence from studies in Japan, the recommended amount of physical activity in the Japanese guidelines is nearly three times higher than for other Western countries and in the WHO guidelines.^10^^,^^15^^,^^16^ Although substantial international evidence supports the health risk of sedentary behavior, these findings may not be directly generalizable to Japanese population due to important contextual differences, such as physical activity patterns, dietary habits, lifestyle, genetic prepositions, and the prevalence of chronic diseases. These distinctions underline the importance of synthesizing country-specific evidence to inform relevant public health recommendations. Therefore, a systematic review of existing evidence from Japan on sedentary behavior is also warranted. As the evidence on prospective associations of sedentary behavior with health outcomes in the Japanese population continues to grow, it is critical to clarify existing research gaps to consider evidence needed to underpin future public health guidelines for Japan.

No formal review has yet addressed the scientific evidence supporting the newly introduced sedentary behavior guidelines for adults in Japan. We report a systematic review of prospective cohort studies examining the relationships of sedentary behavior with health outcomes among Japanese adults aged 18 years or older.

## METHODS

The present systematic review was conducted between March and April 2024 as well as June 2025, following the Preferred Reporting Items for Systematic reviews and Meta-Analyses extension for Scoping Reviews (PRISMA-ScR) guidelines^17^ and the JBI methodology for scoping reviews, including the use of the Population, Concept, Context framework for eligibility criteria, a comprehensive search strategy, data charting, and synthesis processes.^18^ Since the primary focus of this review was to identify evidence gaps regarding the prospective associations of sedentary behavior with health outcomes in Japan, it was classified as a scoping review and did not meet the criteria for registration with PROSPERO.

### Research questions

Based on a preliminary literature search and discussions among authors, the study aimed to address the following primary research question: what is the relationship between sedentary behavior and health outcomes in Japanese adults? Additionally, secondary research questions were: what is the quantity and quality of the existing evidence on this relationship; and what are the knowledge gaps that future research should address?

### Study inclusion criteria

The review included studies based on specific eligibility criteria regarding the population, exposures, outcomes, study designs, and publication status.

#### Population

The target population consisted of apparently healthy Japanese adults aged 18 years or older who did not report the health outcomes of interest at baseline. Ineligible for inclusion were studies of children and adolescents (<18 years) and studies of special populations, including pregnant individuals, residents of long-term care facilities, and clinical patients. Studies were also excluded if their study population consisted solely of participants with specific diseases, such as prospective cohort studies limited to those with pre-existing CVDs or cancer survivors. In clinical populations with pre-existing conditions, daily behaviors, including sedentary behavior, may be significantly altered due to medical restrictions or disease-related limitations. As a result, the associations observed in these populations may not accurately reflect those in the general population, potentially compromising the applicability of the findings to public health guidelines aimed at the broader adult population.

#### Exposures

The primary exposure was sedentary behavior in free-living situations, assessed either using objective measurement devices (eg, accelerometer or inclinometer) or self-report questionnaires. Sedentary behaviors could include total, occupational, transportation-related, leisure, and screen-based (eg, TV viewing, smartphone, PC and tablet using) sedentary time. Studies examining the patterns of sedentary behavior (eg, frequency, timing, and duration of breaks and sedentary bouts) were also included.

#### Outcomes

The review focused on any health outcomes relevant to adults, including older adults (eg, health-related quality of life, all-cause mortality, disease-specific mortality and incidence, risk factors and biomarkers of non-communicable diseases, physical and cognitive function and disability, depression and anxiety, fatigue, and joint and musculoskeletal pain).^19^ Accident and injury and other health behaviors as outcomes (eg, sleep, physical activity) were excluded.

#### Study designs

Only longitudinal observational studies, including prospective and retrospective cohort studies, were targeted. Cross-sectional studies were excluded because their design does not allow for the assessment of temporal sequence between exposure and outcome, which is essential for inferring potential causality. Studies examining the influence of sedentary behavior on health outcomes independently of and in combination with MVPA were also included.

#### Publication status and language

Only peer-reviewed journal articles published in English between January 1, 2000 and December 31, 2023 were eligible for inclusion because studies on this topic began to emerge during this period.^20^

### Data sources and searches

A systematic search was conducted using the electronic bibliographic databases PubMed, Web of Science, CINAHL, and MEDLINE to identify relevant studies. The initial search was performed during the week of March 11, 2024, and an updated search was conducted during the week of April 22, 2024. Four sets of search terms were used: sedentary behavior (eg, sedentary behavior, sitting, television viewing, and screen time), the longitudinal design (eg, cohort, prospective, and retrospective), study population inclusion (Japanese), and exclusion (eg, children, youth, and adolescents). A hand-search of the reference lists of eligible studies was also performed to identify additional relevant literature. The complete list of search terms and syntax used for the search is presented in eTable 1. A supplementary literature search was conducted in two Japanese databases (Ichushi Web and CiNii Articles) on June 3, 2025. The search terms were adapted to the structure of each database and were aligned conceptually with the English-language search strategy. The complete list of search terms and syntax used for the supplementary search is presented in eTable 2.

### Study selection

Following the removal of duplicates using EndNote Reference Manager (version 20, Clarivate Japan, Tokyo), two authors (AS, KO) independently screened the titles and abstracts of identified articles to determine their eligibility for full-text review. Articles that were considered potentially relevant were obtained in full text and reviewed by the same authors. Discrepancies in study selection were resolved through discussion, and a third author (KI) was consulted if a consensus could not be reached.

### Data extraction

Two authors (AS, KO) independently extracted information from eligible studies, including name of first author, year of publication, study design or cohort name, sample size, age of participants, sex distribution, duration of follow-up, number of outcome cases, assessment methods for outcomes and sedentary behavior, covariates included in the analyses, effect estimates, and 95% confidence intervals (CIs) of outcomes. A third author (KI) cross-checked all extracted data for accuracy and consistency.

### Quality assessment

The quality of the studies included was evaluated using the Quality Assessment Tool for Observational Cohort and Cross-Sectional Studies.^21^ This tool comprises a 14-item checklist assessing the following criteria: 1) clearly stated research question; 2) specific study population; 3) rate of participation of eligible persons; 4) subject selection process; 5) justification of sample size; 6) exposure measured prior to outcome(s); 7) suitable timeframe between exposure to outcome; 8) levels of exposure; 9) exposure measures clearly defined; 10) exposure(s) assessed more than once over time; 11) outcome measures being valid, reliable, and consistent; 12) blinding of outcome assessors; 13) loss to follow-up reported; and, 14) adjustment for key confounders. Items could be scored ‘yes’ (1) and ‘no’, ‘cannot determine (CD)’, ‘not applicable (NA)’, or ‘not reported (NR)’ (0). In addition, studies were categorized as good, fair, or poor quality based on the general guidelines for determining overall quality ratings.^21^ Studies were rated as good if it had a low risk of bias, minimal limitations, and used reliable methods for measurement and follow-up. A fair rating was assigned to studies with some limitations or potential sources of bias, but without major flaws that would affect the overall findings. Studies were rated as poor when they had significant limitations or a high risk of bias that reduced confidence in the results.^21^ Two authors (AS, KO) independently conducted the quality assessment, and any discrepancies were resolved through discussion with a third author (KI).

### Presentation of the results

Results were narratively summarized and presented in a tabular format.

## RESULTS

### Study selection

**Figure 1.**
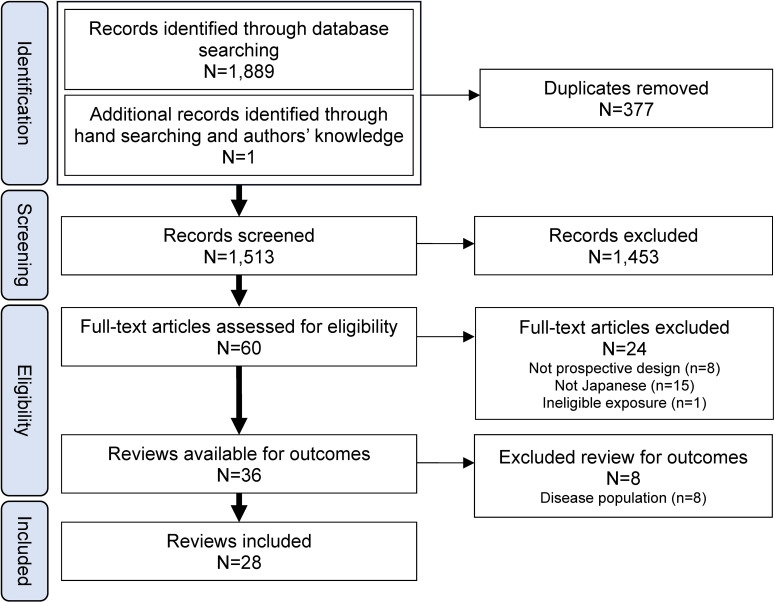
PRISMA flow diagram

The initial search identified 1,889 studies, with one additional study included based on authors’ knowledge. After deduplication, the 1,890 articles were reduced to 1,513. Title and abstract screening excluded 1,453 articles, leaving 60 for full-text review. Of these, 32 did not meet eligibility criteria, resulting in 28 studies for analysis (Figure 1). A list of excluded full texts and reasons can be found in eTable 3.

### Study characteristics

A summary of study characteristics and results is presented in Table 1. The 28 studies, published between 2008 and 2023, were primarily post-2013, with one exception. Regarding the study population, eight studies focused on older adults (29%), 15 on middle-aged and older adults (54%), one on adults in general (≥20 years), and four on working adults. Most studies included both genders, with three only on women. Sixteen studies were based on large cohort data (>10,000 participants) from the Japan Collaborative Cohort Study (JACC study), Japan Public Health Center-based Prospective Study (JPHC study), Japan-Multi-Institutional Collaborative Cohort Study (J-MICC study), and Kyoto-Kameda Study; nine followed 1,000–5,000 participants, and three had ≤500 participants. In terms of sampling, most studies enrolled community-dwelling adults, with two studies focusing on company employees and one using an internet-based sample. Follow-up durations varied: ten studies spanned 15–20 years and eight studies followed ≤3 years (3–4.9 years: *n* = 4; 5–9.9 years: *n* = 3; 10–14.9 years: *n* = 3).

**Table 1.** Summary of the prospective cohort studies on sedentary behavior and health outcomes in Japan

Study	Study Sample	Study Design	Follow-up Duration	Outcome Measure	Sitting Measure	Confounders Measured	Outcome Cases	Summary of Results [effect estimates, and 95% confidence intervals (CIs)]
Inoue et al (2008) JPHC study	83,034 individuals (M = 39,183; W = 43,851) Aged 45–74 years at follow-up survey	Cohort	Mean 8.7 years (725,071 person-years)	Mortality (all-cause)	Self-reported overall ST <3, 3 to 8, ≥8 h/d (ordinal scale)	Age, area, occupation, history of diabetes, smoking status, alcohol intake status, BMI, total energy intake, heavy physical work or strenuous exercise, walking or standing hs, and leisure-time sports or physical exercise	4,564 deaths (M = 3,098; W = 1,466)	HR (95% CI) for all-cause mortality: <3 h/d = 1.00 (ref) 3–8 h/d = 1.02 (0.95–1.11) M, 0.95 (0.85–1.06) W ≥8 h/d = 1.18 (1.04–1.35) M^a,b^, 1.10 (0.82–1.25) W

Ukawa et al (2013) JACC study	54,258 individuals (M = 23,090 men; W = 31,168) w/o a history of cancer at baseline Aged 40–79 years at baseline	Cohort	Median 15.6 years (720,883 person-years)	Incidence (lung cancer)	Self-reported TV time <2, 2 to 4, ≥4 h/d	Age, BMI, education, marital status, alcohol drinking, smoking status, intake of green leafy vegetables, oranges, and fruits other than oranges	798 diagnoses (M = 598; W = 200)	HR (95% CI) for lung cancer incidence: <2 h/d = 1.00 (ref) 2–4 h/d = 1.24 (0.98–1.60) M, 1.11 (0.76–1.67) W ≥4 h/d = 1.36 (1.04–1.80) M^b^, 1.03 (0.67–1.62) W

Ukawa et al (2014) JACC study	69,752 individuals (M = 28,642; W = 41,110) w/o a history of liver disease, cancer, stroke, or MI at baseline and those who died within the first 5 years of follow-up Aged 40–79 years at baseline	Cohort	Median 19.4 years (1,190,482 person-years)	Mortality (liver cancer)	Self-reported TV time <2, 2 to 4, ≥4 h/d	Age, study area, smoking status, alcohol consumption, daily consumption of coffee, BMI, educational level, marital status, and a history of diabetes mellitus, gallbladder diseases, and blood transfusion	267 deaths (M = 163; W = 104)	HR (95% CI) for liver cancer mortality: <2 h/d = 1.00 (ref) 2–4 h/d = 0.98 (0.70–1.38) T, 0.93 (0.62–1.45) M, 1.08 (0.63–1.95) W ≥4 h/d = 1.20 (0.82–1.77) T, 1.23 (0.76–2.02) M, 1.13 (0.62–2.13) W Joint associations with WT ≥4 h/d ST/≤0.5 h/d WT = 1.00 (ref), ≥4 h/d ST/>0.5 h/d WT = 0.64 (0.40–1.02) 2–4 h/d ST/≤0.5 h/d WT = 0.68 (0.42–1.11), 2–4 h/d ST/>0.5 h/d WT = 0.58 (0.39–0.89)^b^ <2 h/d ST/≤0.5 h/d WT = 0.71 (0.36–1.34), <2 h/d ST/>0.5 h/d WT = 0.58 (0.35–0.98)^b^

Ukawa et al (2015) JACC study	76,688 individuals (M = 33,414; W = 43,274) w/o a history of cancer, stroke, MI, or tuberculosis at baseline Aged 40–79 years at baseline	Cohort	Median 19.4 years	Mortality (COPD-related)	Self-reported TV time <2, 2 to 4, ≥4 h/d	Age, study area, smoking status, BMI, education, marital status, alcohol drinking, exercise time	278 deaths (M = 244; W = 34)	HR (95% CI) for COPD–related mortality: <2 h/d = 1.00 (ref) 2–4 h/d = 1.40 (0.92–2.14) M, 1.03 (0.40–2.75) W ≥4 h/d = 1.63 (1.04–2.55) M^a,b^, 0.84 (0.29–2.48) W

Ikehara et al (2015) JACC study	85,899 individuals (M = 35,959; W = 49,940) w/o a history of CVD and cancer at baseline Aged 40–79 years at baseline	Cohort	Median 19.2 years (1,398,591 person-years)	Mortality (Stroke, CAD, total CVD)	Self-reported TV time <2, 2, 3, 4, 5, ≥6 h/d	BMI, smoking, ethanol intake, education level, hs of sport, hs of walking, sleep duration, perceived mental stress, presence of job, frequency of fresh fish intake and depressive symptoms	2,553 deaths from stroke (M = 1,302, W = 1,251) 1,206 deaths from CAD (M = 692, W = 514) 5,835 deaths from CVD (M = 3,002, W = 2,833)	HR (95% CI) for stroke mortality: <2 h/d = 1.00 (ref), 2 h/d = 1.06 (0.93–1.19), 3 h/d = 0.98 (0.87–1.11) 4 h/d = 0.89 (0.76–1.03), 5 h/d = 1.06 (0.90–1.23), ≥6 h/d = 1.15 (0.92–1.32) 1-h increment = 1.01 (0.98–1.03) HR (95% CI) for CAD mortality: <2 h/d = 1.00 (ref), 2 h/d = 1.01 (0.84–1.21), 3 h/d = 0.92 (0.77–1.11) 4 h/d = 1.00 (0.81–1.24), 5 h/d = 1.16 (0.92–1.45), ≥6 h/d = 1.24 (0.96–1.61) 1-h increment = 1.03 (1.00–1.07)^b^ HR (95% CI) for total CVD mortality: <2 h/d = 1.00 (ref), 2 h/d = 1.00 (0.92–1.08), 3 h/d = 0.95 (0.88–1.04) 4 h/d = 0.96 (0.87–1.05), 5 h/d = 1.03 (0.93–1.14), ≥6 h/d = 1.14 (1.02–1.28)^b^ 1-h increment = 1.01 (1.00–1.03)^b^

Kikuchi et al (2015) JPHC study	36,516 community-dwelling workers w/o history of cancer or CVD Aged 50–74 years at baseline	Cohort	Mean 10.1 years (368,120 person-years)	Mortality (all-cause)	Self-reported occupational ST <1, 1 to 3, ≥3 h/d (ordinal scale)	Age, sex, public health centres, smoking, drinking, BMI, diabetes history, walk time at work, strenuous work time, MVPA in leisure time, and hypertension	2,209 deaths	HR (95% CI) for all-cause mortality: Overall <1 h/d = 1.00 (ref) 1–3 h/d = 1.00 (0.88–1.14) M, 1.06 (0.84–1.33) M ≥3 h/d = 0.97 (0.86–1.09) M, 1.15 (0.92–1.42) M Primary industry <1 h/d = 1.00 (ref) 1–3 h/d = 1.16 (0.94–1.43) M, 1.01 (0.73–1.42) W ≥3 h/d = 1.23 (1.00–1.51) M^b^, 1.34 (0.97–1.84) W Secondary or tertiary industry <1 h/d = 1.00 (ref) 1–3 h/d = 0.91 (0.77–1.08) M, 1.09 (0.80–1.51) W ≥3 h/d = 0.87 (0.75–1.01) M, 1.03 (0.77–1.39) W

Shirakawa et al (2016) JACC study	86,024 individuals (M = 36,006; W = 50,018) w/o a history of cancer, stroke, MI, or pulmonary embolism at baseline Aged 40–79 years at baseline	Cohort	Median 19.2 years (1,398,570 person-years)	Mortality (pulmonary embolism)	Self-reported TV time <2.5, 2.5 to 4.9, ≥5.0 h/d	Age, sex, BMI, history of hypertension, history of diabetes mellitus, smoking status, perceived mental stress, educational level, walking activity, and sports activity	59 deaths	HR (95% CI) for pulmonary embolism mortality: <2.5 h/d = 1.00 (ref), 2.5–4.9 h/d = 1.7 (0.9–3.0), >5 h/d = 2.5 (1.2–5.3)^b^ 2-h increment = 1.4 (1.0–1.8)^b^

Honda et al (2016) Ryobi Health Survey	430 office workers (M = 372; W = 58) w/o metabolic syndrome at baseline Aged 40–64 years	Cohort	Median 3 years	Incidence (metabolic syndrome)	Device-measured SB (min/d) Total ST (≥1-min bout), Non-prolonged ST (<30 min bout), Prolonged ST (≥30 min-bout)	Age, sex, education, smoking, family income, MVPA, and waist circumference	83 (M = 76, W = 7) developed metabolic syndrome	HR (95% CI) for metabolic syndrome incidence: Total ST Q1 = 1.00 (ref), Q2 = 1.50 (0.73–3.09), Q3 = 1.76 (0.87–3.55), Q4 = 1.55 (0.70–3.43) Non-prolonged ST Q1 = 1.00 (ref), Q2 = 0.79 (0.42–1.48), Q3 = 1.09 (0.59–2.03), Q4 = 1.08 (0.57–2.02) Prolonged ST Q1 = 1.00 (ref), Q2 = 3.03 (1.42–6.49)^c^, Q3 = 2.25 (1.03–4.92)^b^, Q4 = 2.90 (1.30–6.44)^c^

Kitayuguchi et al (2016) published in Japanese	1,890 community-dwelling adults aged 60–70 years	Cohort	1 years	Incidence (fall)	Self-reported total ST (h/d) 0–119, 120–179, 180–269, 270–419, 270–419, ≥420 min/d	Age, sex, BMI, community, y of education, self-rated health, depressive symptoms, smoking habit, chronic disease history, MVPA, (Model 2: medication use and consultation with physical for chronic low back pain; Model 3: medication use and consultation with physical for chronic knee pain)	163 experienced falls	OR (95% CI) for fall Model 1 (major sociodemographic and health-related covariates) 0–119 min/d = 1.00 (ref), 120–179 min/d = 1.74 (0.90–3.38), 180–269 min/d = 1.59 (0.85–2.69) 270–419 min/d = 1.61 (0.81–3.20), ≥420 min/d = 1.91 (1.00–3.66) Model 2 (Model 1 and chronic low back pain related covariates) 0–119 min/d = 1.00 (ref) 120–179 min/d = 1.73 (0.90–3.35), 180–269 min/d = 1.57 (0.84–2.93) 270–419 min/d = 1.60 (0.80–3.18), ≥420 min/d = 1.90 (0.99–3.65) Model 3 (Model 1 and chronic knee pain related covariates) 0–119 min/d = 1.00 (ref) 120–179 min/d = 1.76 (0.91–3.43), 180–269 min/d = 1.57 (0.84–2.94) 270–419 min/d = 1.61 (0.81–3.19), ≥420 min/d = 1.96 (1.02–3.79)^b^

Tsutsumimoto et al (2017) OSHPE study	3,503 older individuals w/o any support or care by the Japanese public long-term care system, a history of Alzheimer’s disease, stroke, Parkinson’s disease, or depression disorders, Aged ≥65 years at baseline	Cohort	15 months	Incidence (clinical depressive symptoms)	Self-reported total ST <240, 240 to 480, ≥480 min/d	Age, sex, education, smoking status, alcohol consumption, living	232 developed depressive symptoms	OR (95% CI) for clinical depressive symptom incidence: <240 min/d = 1.00 (ref) 240–480 min/d = 1.61 (1.09–2.38)^b^ ≥480 min/d = 1.64 (1.02–2.64)^b^

Ukawa et al (2018) JACC study	34,758 female individuals w/o a history of cancer at baseline Aged 40–79 years at baseline	Cohort	Median 19.4 years	Incidence (ovarian cancer)	Self-reported TV time <2, 2 to 2.9, 3 to 3.9, 4 to 4.9, ≥5 h/d	Age, BMI, educational level, smoking status, alcohol drinking status, average daily sleeping time, average daily walking time, age at menarche, age at menopause, parity, use of hormone therapy ever, family history of breast, ovarian, or prostate cancer	59 diagnoses of ovarian cancer	HR (95% CI) for ovarian cancer incidence: <2 h/d = 1.00 (ref) 2–2.9 h/d = 1.03 (0.70–1.50), 3–3.9 h/d = 1.18 (0.82–1.70) 4–4.9 h/d = 0.81 (0.54–1.21), ≥5 h/d = 2.15 (1.54–2.99)^b^

Ikehara et al (2019) JACC study	25,240 participants (M = 9,786; W = 15,454) w/o a history of diabetes, stroke, CAD, or cancer at baseline Aged 40–79 years at baseline	Cohort	5 years	Incidence (type 2 diabetes) ^*^self-reported physician-diagnosis	Self-reported TV time <2, 2, 3, 4, ≥5 h/d	Age, sex, alcohol consumption, history of hypertension, smoking status, hs of exercise, stress, educational level, unemployed, and sleep duration, walking time, BMI	778 diagnoses (M = 397, W = 381)	HR (95% CI) for type 2 diabetes incidence: <2 h/d = 1.00 (ref) 2 h/d = 0.93 (0.74–1.18) T, 0.84 (0.61–1.16) M, 1.07 (0.75–1.52) W 3 h/d = 1.16 (0.92–1.46) T, 1.09 (0.80–1.48) M, 1.27 (0.90–1.78) W 4 h/d = 1.09 (0.83–1.44) T, 1.10 (0.76–1.61) M, 1.06 (0.70–1.61) W ≥5 h/d = 1.30 (1.00–1.70) T^a,b^ 1.06 (0.71–1.59) M, 1.51 (1.03–2.19) W^a,b^ Combined associations with WT ≥5 h TV/<1 h WT = 1.00 (ref) ≥5 h TV/≥1 h WT = 0.98 (0.68–1.40) T, 1.19 (0.66–2.15) M, 0.84 (0.53–1.33) W <5 h TV/<1 h walking = 0.86 (0.66–1.12) T 1.11 (0.72–1.70) M, 0.73 (0.52–1.03) W <5 h TV/≥1 h walking = 0.72 (0.55–0.94) T^b^, 0.88 (0.57–1.36) M, 0.64 (0.45–0.90) W^b^

Lee et al (2019) OSHPE study	4,457 community-dwelling older individuals w/o any support or care by the Japanese public long-term care system, a history of Parkinson’s disease, stroke, dementia Aged ≥65 years at baseline	Cohort	4 years	Incidence (functional disability certified by long-term care insurance system)	Self-reported total ST <8, ≥8 h/d	Age, sex, education level, BMI, muscle mass, MMSE score, GDS, smoking habit, drinking habits, medication, total protein, triglyceride, grip strength, gait speed, regular exercise, hypertension, heart disease, osteoporosis, diabetes, and cancer	461 incidence of functional disability (certified as needing long-term care insurance system)	HR (95% CI) for disability incidence: <8 h/d eGFR >60 = 1.00 (ref), eGFR 45–59 = 0.94 (0.77–1.31), eGFR <45 = 1.45 (0.94–2.23) ≥8 h/d eGFR >60 = 1.00 (ref), eGFR 45–59 = 1.34 (0.82–2.18), eGFR <45 = 4.37 (2.02–9.44)^d^

Cao et al (2019) JACC study	33,276 (17,568 premenopausal, and 15,708 postmenopausal) women w/o previous diagnosis of breast cancer Aged 40–79 years at baseline	Cohort	Median 16.8 years (607,295 person years)	Incidence (breast cancer)	Self-reported TV time <1.5, 1.5 to <3.0, 3.0 to <4.5, ≥4.5 hr/d	Age, age of menarche, BMI, parity, family history of breast cancer, education level, married status, dtime napping, sleep duration, mental stress, alcohol intake, hormone use, smoking status, history of diabetes (for postmenopausal women adjusted further for age of menopause, type of menopause), sport time and walking time	247 new cancer cases (170 premenopausal; 77 postmenopausal)	HR (95% CI) for breast cancer incidence: Total <1.5 h/d = 1.00 (ref), 1.5 to 3.0 h/d = 0.89 (0.59–1.34), 3 to 4.5 h/d = 1.19 (0.82–1.74), ≥4.5 h/d = 1.45 (0.91–2.32) Premenopausal <1.5 h/d = 1.00 (ref), 1.5 to 3.0 h/d = 0.85 (0.58–1.43), 3 to 4.5 h/d = 0.91 (0.58–1.43) ≥4.5 h/d = 1.34 (0.76–2.36) Postmenopausal <1.5 h/d = 1.00 (ref) 1.5 to 3.0 h/d = 1.10 (0.42–2.88), 3 to 4.5 h/d = 2.54 (1.11–5.80)^b^ ≥4.5 h/d = 2.37 (0.92–6.10)^a^

Sakaue et al (2020)	1,680 individuals (M = 693, W = 987) Aged 40–95 years	Cohort	Mean 15.9 years	Mortality (all-cause, CVD, cancer)	Self-reported occupational ST never (1), seldom (2), sometimes (3), often (4) (4 likert scale)	Age, sex, BMI, total cholesterol, systolic blood pressure, total physical activity index	397 deaths (M = 224, W = 173)	HR (95% CI) for all-cause morality: Occupational sitting = 1.11 (1.01–1.22) T^b^, 1.28 (1.13–1.46) M^d^, 1.02 (0.88–1.17) W HR (95% CI) for cancer morality: Occupational sitting = 1.10 (0.92–1.30) HR (95% CI) for CVD morality: Occupational sitting = 1.39 (1.01–1.91)^b^

Ihira et al (2020) JPHC study	33,307 individuals (M = 20,030; W = 13,277) w/o history of cancer, CVD, or physical limitation at baseline Aged 50–79 years at baseline	Cohort	Mean 10.2 years (373,809 person-years)	Incidence (cancer: total, stomach, colorectal, colon, rectum, liver, pancreas, lung, kidney, bladder, prostate, other)	Self-reported occupational ST <1, 1 to <3, 3 to <5, 5 to <7, ≥7 h/d (ordinal scale)	Age, area, history of diabetes, smoking status, alcohol intake status, BMI, coffee, walking time at work, strenuous time at work, MVPA time in leisure time, type of job and total working hs	3,807 new cancer cases (M = 2,842; W = women)	HR (95% CI) for total cancer incidence: <1 hr/d = 1.02 (0.93–1.14) M, 1.15 (0.97–1.38) W 1 to <3 h/d = 1.00 (ref) 3 to <5 h/d = 1.08 (0.96–1.21) M, 1.38 (1.15–1.66) W 5 to <7 h/d = 1.03 (0.90–1.17) M, 1.09 (0.87–1.36) W ≥7 h/d = 1.12 (0.99–1.26) M, 1.08 (0.87–1.33) W HR (95% CI) for esophagus cancer incidence: <1 hr/d = 0.92 (0.55–1.53) M, 1.64 (0.25–10.75) W 1 to <3 h/d = 1.00 (ref) 3 to <5 h/d = 0.72 (0.37–1.42) M, 2.38 (0.35–16.45) W 5 to <7 h/d = 0.65 (0.29–1.43) M, – W ≥7 h/d = 1.05 (0.58–1.87) M, – W HR (95% CI) for stomach cancer incidence: <1 hr/d = 0.99 (0.79–1.25) M, 0.89 (0.54–1.46) W 1 to <3 h/d = 1.00 (ref) 3 to <5 h/d = 1.09 (0.84–1.41) M, 1.03 (0.61–1.74) W 5 to <7 h/d = 1.04 (0.78–1.40) M, 1.35 (0.78–2.36) W ≥7 h/d = 1.08 (0.82–1.41) M, 1.03 (0.59–1.81) W HR (95% CI) for colorectal cancer incidence: <1 hr/d = 0.99 (0.78–1.25) M, 1.22 (0.84–1.78) W 1 to <3 h/d = 1.00 (ref) 3 to <5 h/d = 1.26 (0.98–1.64) M, 1.26 (0.85–1.87) W 5 to <7 h/d = 1.02 (0.76–1.37) M, 1.36 (0.87–2.11) W ≥7 h/d = 1.17 (0.91–1.52) M, 0.94 (0.58–1.53) W
								HR (95% CI) for colon cancer incidence: <1 hr/d = 0.89 (0.65–1.21) M, 1.20 (0.77–1.85) W 1 to <3 h/d = 1.00 (ref) 3 to <5 h/d = 1.37 (1.00–1.87) M+, 1.27 (0.81–2.00) W 5 to <7 h/d = 1.13 (0.79–1.61) M, 1.24 (0.74–2.09) W ≥7 h/d = 1.19 (0.87–1.65) M, 0.91 (0.52–1.60) W HR (95% CI) for rectum cancer incidence: <1 hr/d = 1.18 (0.80–1.73) M, 1.27 (0.60–2.68) W 1 to <3 h/d = 1.00 (ref) 3 to <5 h/d = 1.07 (0.68–1.68) M, 1.20 (0.52–2.76) W 5 to <7 h/d = 0.80 (0.46–1.39) M, 1.76 (0.74–4.16) W ≥7 h/d = 1.13 (0.73–1.75) M, 1.00 (0.37–2.69) W HR (95% CI) for liver cancer incidence: <1 hr/d = 0.84 (0.51–1.39) M, 0.66 (0.24–1.84) W 1 to <3 h/d = 1.00 (ref) 3 to <5 h/d = 1.18 (0.70–1.98) M, 0.58 (0.16–2.13) W 5 to <7 h/d = 1.00 (0.54–1.83) M, 0.31 (0.04–2.49) W ≥7 h/d = 1.54 (0.92–2.58) M, 0.30 (0.04–2.20) W HR (95% CI) for pancreas cancer incidence: <1 hr/d = 1.17 (0.61–2.28) M, 1.36 (0.53–3.46) W 1 to <3 h/d = 1.00 (ref) 3 to <5 h/d = 1.53 (0.74–3.16) M, 2.10 (0.82–5.37) W 5 to <7 h/d = 1.82 (0.86–3.84) M, 2.12 (0.75–5.96) W ≥7 h/d = 2.25 (1.17–4.34) M^a,b^, 0.90 (0.26–3.07) W
								HR (95% CI) for lung cancer incidence: <1 hr/d = 1.12 (0.84–1.48) M, 2.17 (1.11–4.26) W 1 to <3 h/d = 1.00 (ref) 3 to <5 h/d = 1.30 (0.94–1.79) M, 2.63 (1.30–5.30) W 5 to <7 h/d = 1.17 (0.82–1.68) M, 1.26 (0.48–3.35) W >7 h/d = 1.07 (0.75–1.51) M, 2.80 (1.33–5.90) W^a,b^ HR (95% CI) for kidney cancer incidence: <1 hr/d = 1.35 (0.68–2.69) M, 1.73 (0.29–10.44) W 1 to <3 h/d = 1.00 (ref) 3 to <5 h/d = 1.11 (0.49–2.51) M, 3.40 (0.61–18.91) W 5 to <7 h/d = 0.59 (0.19–1.81) M, 4.15 (0.66–26.11) W >7 h/d = 1.04 (0.44–2.47) M, – W HR (95% CI) for bladder cancer incidence: <1 hr/d = 0.86 (0.51–1.44) M, 0.83 (0.20–3.50) W 1 to <3 h/d = 1.00 (ref) 3 to <5 h/d = 0.68 (0.34–1.33) M, 0.54 (0.10–2.81) W 5 to <7 h/d = 0.92 (0.46–1.82) M, 1.42 (0.33–6.13) W >7 h/d = 0.56 (0.27–1.17) M, 0.79 (0.14–4.27) W HR (95% CI) for prostate cancer incidence (only M): <1 hr/d = 1.09 (0.86–1.37), 1 to <3 h/d = 1.00 (ref), 3 to <5 h/d = 0.92 (0.70–1.22) 5 to <7 h/d = 0.89 (0.65–1.22), >7 h/d = 1.10 (0.84–1.45)
								HR (95% CI) for breast cancer incidence (only W): <1 hr/d = 1.21 (0.79–1.84), 1 to <3 h/d = 1.00 (ref), 3 to <5 h/d = 1.39 (0.89–2.15), 5 to <7 h/d = 1.04 (0.61–1.78), ≥7 h/d = 1.11 (0.69–1.81) HR (95% CI) for ovarian cancer incidence (only W): <1 hr/d = 1.11 (0.38–3.11), 1 to <3 h/d = 1.00 (ref), 3 to <5 h/d = 0.93 (0.27–3.22), 5 to <7 h/d = –, ≥7 h/d = 1.51 (0.48–4.72) HR (95% CI) for endometrial cancer incidence (only W): <1 hr/d = 1.37 (0.66–2.83), 1 to <3 h/d = 1.00 (ref), 3 to <5 h/d = 0.99 (0.41–2.40), 5 to <7 h/d = 1.41 (0.58–3.47), ≥7 h/d = 0.49 (0.15–1.52) HR (95% CI) for other cancer incidence: <1 hr/d = 1.02 (0.77–1.33) M, 0.97 (0.66–1.42) W 1 to <3 h/d = 1.00 (ref) 3 to <5 h/d = 1.07 (0.79–1.47) M, 1.53 (1.06–2.21) W 5 to <7 h/d = 1.29 (0.93–1.78) M, 0.68 (0.39–1.18) W ≥7 h/d = 1.01 (0.73–1.39) M, 1.16 (0.75–1.78) W

Miyata et al (2021) JACC study	33,801 women w/o history of cancer or uterine surgery Aged 40–79 years at baseline	Cohort	Median 14.8 years (455,534 person-years)	Incidence (endometrial cancer)	Self-reported occupational activity mainly sitting, mainly standing, moving Self-reported TV time <1, 1 to <2, 2 to <3, 3 to <4, ≥4 h/d	Age, BMI, weight change since age 20, history of diabetes, history of hypertension, age at menarche, menstrual presence, parity, smoking status, alcohol consumption, occupational activity, hs of physical exercise, walking, and television viewing	79 new cancer cases	HR (95% CI) for endometrial cancer incidence: Occupational activity mainly sitting = 1.00 (ref), mainly standing = 0.79 (0.39–1.59), moving = 0.46 (0.22–0.97)^a,b^ TV time <1 h/d = 0.50 (0.13–1.92), 1 to <2 h/d = 1.00 (ref), 2 to <3 h/d = 1.07 (0.54–2.15), 3 to <4 h/d = 0.72 (0.34–1.53), ≥4 h/d = 1.05 (0.51–2.15)

Watanabe & Kawakami (2021)	231 workers w/o major depression episode or official absent due to mental health problem in the past 12 months	Cohort	12 months (1,621 person-months)	Incidence (major depression episode)	Self-reported ST at work (h/d) <9.5 h/d 9.5+ h/d	Age, sex, educational status, marital status, household income, drinking, smoking, working hs, physical activity, propensity scores of covariates	6 incidents of major depression episodes	HR (95% CI) for incidence of major depression episode <9.5 h/d = 1.00 (ref) 9.5+ h/d = 2.11 (0.42–10.22)

Koohsari et al (2021)	1086 company workers who registered Japanese internet research service company Aged 20–59 years at baseline	Cohort	18 months	self-reported fatigue score change (subjective fatigue, concentration, motivation, physical activity)	Self-reported domain-specific ST (work, car driving or riding, PT use, TV viewing, PC use, other leisure purposes) workd, usual week (workday+non-workday)	Age, sex, marital status, education attainment, household income, baseline fatigue	not applicable	Unstandardized regression coefficient (β) (95% CI) Workday Subjective fatigue Car = −0.05 (−0.55–0.44), PT = −0.23 (−0.82–0.36), Work = 0.02 (−0.13–0.16), TV = 0.07 (−0.27–0.40), PC = 0.05 (−0.25–0.35), Other = 0.30 (−0.17–0.76), Total = 0.03 (−0.17–0.76) Concentration Car = −0.04 (−0.30–0.22), PT = −0.08 (−0.38–0.23), Work = 0.07 (−0.01–0.14), TV = 0.06 (−0.11–0.23), PC = 0.13 (−0.28–0.03), Other = 0.02 (−0.22–0.26), Total = 0.02 (−0.03–0.08) Motivation Car = −0.01 (−0.25–0.24), PT = −0.29 (0.00–0.36)^b^, Work = 0.02 (−0.05–0.10), TV = 0.02 (−0.16–0.13), PC = −0.02 (−0.18–0.14), Other = 0.40 (0.18–0.62)^b^, Total = 0.04 (−0.01–0.09) Physical activity Car = 0.03 (−0.16–0.22), PT = 0.04 (−0.19–0.26), Work = 0.06 (0.00–0.12)^b^, TV = 0.04 (−0.09–0.17), PC = 0.03 (−0.09–0.14), Other = 0.30 (−0.06–0.29), Total = 0.05 (0.01–0.09)^b^ Total fatigue Car = −0.05 (−0.94–0.85), PT = 0.01 (−1.04–1.06), Work = 0.16 (−0.10–0.42), TV = 0.16 (−0.44–0.76), PC = −0.06 (−0.61–0.48), Other = 0.76 (−0.08–1.59), Total = 0.14 (−0.06–0.33)
								Unstandardized regression coefficient (β) (95% CI) Usual week Subjective fatigue Car = −0.23 (−0.36–0.82), PT = −0.27 (−1.01–0.47), Work = −0.02 (−0.21–0.16), TV = 0.11 (−0.19–0.40), PC = −0.02 (−0.31–0.27), Other = 0.30 (−0.16–0.62), Total = 0.03 (−0.09–0.14) Concentration Car = −0.07 (−0.24–0.37), PT = −0.13 (−0.51–0.26), Work = 0.07 (−0.02–0.17), TV = 0.11 (−0.04–0.26), PC = −0.11 (−0.26–0.04), Other = 0.02 (−0.18–0.22), Total = 0.03 (–0.03–0.09) Motivation Car = 0.09 (−0.20–0.37), PT = –0.31 (−0.05–0.67), Work = 0.03 (−0.06–0.12), TV = 0.03 (−0.11–0.17), PC = 0.03 (−0.11–0.17), Other = 0.26 (0.07–0.45)^b^, Total = 0.06 (0.00–0.11)^b^ Physical activity Car = 0.10 (−0.13–0.32), PT = 0.05 (−0.23–0.33), Work = 0.07 (0.00–0.14)^b^, TV = 0.07 (−0.04–0.18), PC = 0.04 (−0.07–0.15), Other = 0.09 (−0.06–0.24), Total = 0.06 (0.01–0.10)^b^ Total fatigue Car = −0.50 (−0.55–1.56), PT = −0.02 (−1.34–1.31), Work = 0.15 (−0.18–0.48), TV = 0.33 (−0.20–0.85), PC = −0.05 (−0.57–0.46), Other = 0.52 (−0.18–1.21), Total = 0.17 (−0.04–0.36)

Li et al (2021) JACC study	90,834 individuals (M = 38,130, W = 52,704) w/o history of colorectal cancer Aged 40–79 years at baseline	Cohort	Median 19.1 years (1,460,101 person-years)	Mortality (colorectal cancer)	Self-reported TV time <1.5, 1.5 to <3.0, 3.0 to <4.5, ≥4.5 h/d	Age, sex, areas, smoking, drinking, family history of colorectal cancer, education level, frequency of bowel movement, frequency consumption of beef and pork, hs of sport, minutes of waking and BMI	749 deaths (M = 385; W = 364)	HR (95% CI) for colorectal cancer mortality: <1.5 h/d = 1.00 (ref), 1.5 to 3.0 h/d = 1.11 (0.88–1.41), 3 to 4.5 h/d = 1.14 (0.91–1.42) ≥4.5 h/d = 1.33 (1.02–1.73)^a,b^ 1 h-increment = 1.06 (1.01–1.11)^b^ HR (95% CI) for colon cancer mortality: <1.5 h/d = 1.00 (ref), 1.5 to 3.0 h/d = 1.22 (0.92–1.63), 3 to 4.5 h/d = 1.20 (0.91–1.58) ≥4.5 h/d = 1.43 (1.04–1.96)^a,b^ 1 h-increment = 1.07 (1.01–1.13)^b^ HR (95% CI) for right colon cancer mortality: <1.5 h/d = 1.00 (ref), 1.5 to 3.0 h/d = 1.05 (0.60–1.86), 3 to 4.5 h/d = 1.36 (0.81–2.31) ≥4.5 h/d = 1.16 (0.60–2.24) 1 h-increment = 1.08 (0.97–1.20) HR (95% CI) for transverse colon cancer mortality: <1.5 h/d = 1.00 (ref), 1.5 to 3.0 h/d = 2.74 (0.60–12.56), 3 to 4.5 h/d = 2.67 (0.60–11.80) ≥4.5 h/d = 3.55 (0.72–17.46) 1 h-increment = 1.19 (0.97–1.44) HR (95% CI) for left colon cancer mortality: <1.5 h/d = 1.00 (ref), 1.5 to 3.0 h/d = 1.23 (0.87–1.73), 3 to 4.5 h/d = 1.09 (0.78–1.52) ≥4.5 h/d = 1.42 (0.98–2.07) 1 h-increment = 1.06 (0.99–1.13) HR (95% CI) for rectal cancer mortality: <1.5 h/d = 1.00 (ref), 1.5 to 3.0 h/d = 0.90 (0.59–1.36), 3 to 4.5 h/d = 1.01 (0.69–1.50) ≥4.5 h/d = 1.13 (0.69–1.85) 1 h-increment = 1.03 (0.94–1.13)

Koyama et al (2021) J-MICC study	64,456 individuals (M = 29,022, W = 35,434) Aged 35–69 years at baseline	Cohort	Mean 7.7 years (498,670 person-years)	Mortality (all-cause)	Self-reported total ST <5, 5 to <7, 7 to <9, ≥9 h/d	Age, sex, research area, leisure-time-metabolic equivalents, drinking and smoking status, ischemic heart disease, stroke, and history of medication for hypertension, dyslipidemia, and diabetes mellitus.	2,257 deaths (720 hypertension, 454 dyslipidemia, 306 diabetes mellitus)	HR (95% CI) for all-cause mortality: All <5 h/d = 1.00 (ref), 5 to <7 h/d = 1.03 (0.92–1.16), 7 to <9 h/d = 1.21 (1.06–1.36)^b^, ≥9 h/d = 1.54 (1.39–1.71)^b^ 2-h increments in sedentary time = 1.15 (1.11–1.19)^b^ Hypertension <5 h/d = 1.00 (ref), 5 to <7 h/d = 1.08 (0.87–1.33), 7 to <9 h/d = 1.33 (1.06–1.66)^b^, ≥9 h/d = 1.73 (1.43–2.09)^b^ 2-h increments in sedentary time = 1.20 (1.13–1.28)^b^ Dyslipidemia <5 h/d = 1.00 (ref), 5 to <7 h/d = 1.13 (0.87–1.45), 7 to <9 h/d = 1.41 (1.07–1.85)^b^, ≥9 h/d = 1.61 (1.26–2.06)^b^ 2-h increments in sedentary time = 1.18 (1.09–1.27)^b^ Diabetes mellitus <5 h/d = 1.00 (ref), 5 to <7 h/d = 1.57 (1.13–2.17)^b^, 7 to <9 h/d = 1.73 (1.23–2.44)^b^, ≥9 h/d = 2.14 (1.60–2.88)^b^ 2-h increments in sedentary time = 1.27 (1.16–1.40)^b^
								HR (95% CI) for all cause mortality: None of hypertension, dyslipidemia, or diabetes mellitus <5 h/d = 1.00 (ref), 5 to <7 h/d = 1.02 (0.87–1.19), 7 to <9 h/d = 1.13 (0.95–1.34) >9 h/d = 1.45 (1.25–1.67)^b^ 2-h increments in sedentary time = 1.13 (1.16–1.40)^b^ 1 <5 h/d = 1.00 (ref), 5 to <7 h/d = 0.90 (0.72–1.12), 7 to <9 h/d = 1.21 (0.97–1.52), >9 h/d = 1.62 (1.34–1.96)^b^ 2-h increments in sedentary time = 1.18 (1.11–1.26)^b^ 2 <5 h/d = 1.00 (ref), 5 to <7 h/d = 1.31 (0.94–1.82), 7 to <9 h/d = 1.39 (0.97–1.99), >9 h/d = 1.74 (1.28–2.37)^b^ 2-h increments in sedentary time = 1.19 (1.08–1.32)^b^ 3 <5 h/d = 1.00 (ref), 5 to <7 h/d = 2.27 (1.11–4.63)^b^, 7 to <9 h/d = 2.68 (1.32–5.44)^b^, >9 h/d = 3.18 (1.62–6.21)^b^ 2-h increments in sedentary time = 1.42 (1.16–1.73)^b^

Chiba et al (2022) The National Center for Geriatrics and Gerontology-Study of Geriatric Syndrome	3,961 community-dwelling older adults w/o receiving any support or care by long-term care insurance system, history of dementia stroke, Parkinson’s disease, or depression Aged ≥65 years at baseline	Cohort	2 years	Incidence (functional disability certified by long-term care insurance system)	Device-measured SB Total ST (10-min/d)	Age, sex, education level, cognitive function, depression, comorbidity (diabetes, hypertension, hyperlipidemia, heart disease, pulmonary disease, and osteoporosis), number of medication, smoking, alcohol, BMI, grip strength, walking speed wearing time (single-factor and IS models)	142 incidence of functional disability (certified as needing long-term care insurance system)	HR (95% CI) for incidence of disability Single-factor model SB: 1.08 (1.01–1.16)^b^, LPA: 0.94 (0.84–1.05), MVPA: 0.87 (0.77–0.99)^b^ Partition model SB: 1.02 (1.00–1.04)^b^, LPA: 1.02 (0.92–1.05), MVPA: 0.89 (0.78–1.01) IS model SB: dropped, LPA: 0.98 (0.87–1.10), MVPA: 0.87 (0.76–0.99)^b^

Sato et al (2022) Tsuru Longitudinal Study	6,661 older adults living independently in the local community Aged ≥65 years at baseline	Cohort	33 months	Incidence (functional disability certified by long-term care insurance system)	Self-reported total ST (min/d) calculated by summing the time spent doing five distinct activities; reading, conversations with other family members, PC use, TV viewing, other activities that involve lying/sitting positions <190 min/d (short), ≥190 min/d (long) self-reported MVPA time (IPAQ-SV) 0 min/week (Non), 1–299 min/week (short), >300 min/week (long)	Age, sex, educational background, marital status, smoking, drinking, chronic conditions (hypertension, hyperlipidemia, diabetes, stroke, heart disease, arthritis, hip fracture), BMI, nutrition status, self-reported MVPA	517 developed functional disability (certified as needing long-term care insurance system)	HR (95% CI) for incidence of functional disability <190 min/d (short) = 1.00 (ref), ≥190 min/d (long) = 0.86 (0.71–1.03) Non-MVPA/long-SB = 1.00 (ref), Non-MVPA/short-SB = 0.84 (0.64–1.10) Short-MVPA/long-SB = 0.69 (0.25–0.93)^b^, Short-MVPA/short-SB = 0.55 (0.40–0.76)^b^ Long-MVPA/long-SB = 0.49 (0.34–0.68)^b^, Long-MVPA/short-SB = 0.49 (0.34–0.72)^b^

Nemoto et al (2022) Tsuru Longitudinal Study	5,323 community-dwelling older adults in a rural area w/o receiving any support or care by long-term care insurance system Aged ≥65 years at baseline	Cohort	5 years	Incidence (dementia)	Self-reported TV time <1 h, 1 to 3 h, >3 h/d Self-reported book- or newspaper-reading time <10 min (low), 10 to 30 min (moderate), >30 min/d (high) self-reported MVPA (IPAQ-SV) <2.5 METs-h/week (low), 2.5 to 16.0 METs-h/week (moderate), >16 METs-h/week (high)	Age, sex, education level, marital status, living status, employment status, self-rated health, medical conditions (stroke, diabetes, or hypertension), frailty	606 diagnoses of dementia	HR (95% CI) for incidence of dementia Reading time (mentally-active SB) Low = 1.00 (ref) Moderate = 0.83 (0.67–1.03) all, 0.89 (0.68–1.17) low PA, 0.80 (0.52–1.21) moderate PA, 0.44 (0.22–0.85)^b^ high PA High = 0.75 (0.59–0.95)^b^ all, 0.75 (0.54–1.03) low PA, 0.77 (0.49–1.22) moderate PA, 0.46 (0.23–0.93)^b^ high PA TV time (mentally-passive SB) Low = 1.00 (ref) Moderate = 0.98 (0.80–1.19) T, 1.06 (0.82–1.38) low PA, 0.77 (0.54–1.11) moderate PA, 1.23 (0.65–2.32) high PA High = 1.05 (0.81–1.37) T, 1.02 (0.72–1.44) low PA, 1.21 (0.76–1.91) moderate PA, 0.74 (0.27–2.02) high PA Joint impact of PA and mentally active and passive SBs Reading time (mentally-active SB) Low PA/low SB = 1.00 (ref) Low PA/moderate SB = 0.91 (0.69–1.21), Low PA/high SB = 0.74 (0.54–1.03), Moderate PA/low SB = 0.75 (0.50–1.14), Moderate PA/moderate SB = 0.61 (0.44–.083)^b^, Moderate PA/high SB = 0.60 (0.44–0.83)^b^, High PA/low SB = 0.91 (0.50–1.61), High PA/moderate SB = 0.38 (0.24–0.61)^b^, High PA/high SB = 0.38 (0.23–0.62)^b^ TV time (mentally-passive SB) Low PA/high SB = 1.00 (ref) Low PA/moderate SB = 1.06 (0.81–1.40), Low PA/low SB = 1.00 (0.69–1.42), Moderate PA/high SB = 0.81 (0.58–1.14), Moderate PA/moderate SB = 0.64 (0.47–0.87)^b^, Moderate PA/low SB = 1.01 (0.68–1.50), High PA/high SB = 0.51 (0.30–0.88)^b^, High PA/moderate SB = 0.60 (0.40–0.90)^b^, High PA/low SB = 0.38 (0.17–0.87)^b^

Watanabe et al (2022) Kyoto-Kameda Study	10,233 older adults living in Kameda w/o receiving any support or care (level 1–2) by long-term care insurance system, and history of CVD and cancer Aged >65 years	Cohort	Median 5.3 years (51,553 person-years)	Mortality (all-cause)	Self-reported total ST (h/d) Median ST (300 min/d) self-reported MVPA <150 min/week 150+min/week High PA/low SB = PA: 150+min/week and SB: <300 min/d High PA/high SB = PA: 150+week/d and SB: >300 min/d Low PA/low SB = PA: <150 min/week and SB: <300 min/d Low PA/high SB = PA: <150 week/d and SB: >300 min/d	Age, sex, population density, BMI, family structure, economic status, educational attainment, smoking status, alcohol consumption, self-reported health status, sleeping time, medication use, number of chronic disease	1,014 deaths	HR (95% CI) for all-cause mortality: High PA/low SB = 1.00 (ref), High PA/high SB = 0.86 (0.66–1.12) Low PA/low SB = 1.09 (0.88–1.35), Low PA/high SB = 1.36 (1.10–1.67)^b^ Additive interaction = 0.41 (0.12–0.71)^d^ Multiplicative interaction = 1.44 (1.07–1.94)^b^ MVPA 150+min/week = 1.00 (ref), <150 min/week = 1.32 (1.13–1.54) SB <300 min/d = 1.00 (ref), >300 min/d = 1.14 (1.00–1.30)^b^ Restricted cubic spline model of sitting time = a dose-dependent manner up to 420 min (ref: 0 min)

Teramoto et al (2023) JACC study	76,572 individuals (851 stroke survivors, 1,883 MI survivors, 73,838 persons w/o history of stroke or MI) Aged 40–79 years at baseline	Cohort	Median 19.3 years (stroke survivors: 10,279 person-years, MI survivors: 24,636 person-years, person with no history of stroke or MI: 1,222,921 person-years)	Mortality (all-cause, CVD)	Self-reported TV time <3, 3 to 5, 5 to <7, ≥7 h/d	Age, sex, h of exercise, h of walking, history of hypertension, history of diabetes, BMI, smoking, drinking, perceived mental stress, education level, regular employment, dietary intake of vegetable, fish, fruits and soybeans	17,387 deaths (497 stroke survivors, 675 MI survivors, 16,215 persons with no history of stroke or MI)	HR (95% CI) for all-cause mortality: History of stroke <3 h/d = 1.00 (ref), 3–4.9 h/d = 1.18 (0.95–1.48), 5–6.9 h/d = 1.12 (0.86–1.45), ≥7 h/d = 1.61 (1.12–2.32)^a,b^ 2 h-increment = 1.12 (1.03–1.23)^b^ History of MI <3 h/d = 1.00 (ref), 3–4.9 h/d = 0.97 (0.81–1.17), 5–6.9 h/d = 1.40 (1.12–1.76)^b^, ≥7 h/d = 1.44 (1.02–2.03)^a,b^ 2 h-increment = 1.10 (1.01–1.20)^b^ No history of stroke or MI <3 h/d = 1.00 (ref), 3–4.9 h/d = 1.00 (0.96–1.03), 5–6.9 h/d = 1.07 (1.01–1.12)^b^, ≥7 h/d = 1.22 (1.11–1.34)^a,b^ 2 h-increment = 1.04 (1.02–1.06)^b^ HR (95% CI) for CVD mortality: History of stroke <3 h/d = 1.00 (ref), 3–4.9 h/d = 1.28 (0.93–1.77), 5–6.9 h/d = 1.12 (0.76–1.63), ≥7 h/d = 1.77 (1.08–2.91)^b^ 2 h-increment = 1.12 (0.99–1.27) History of MI <3 h/d = 1.00 (ref), 3–4.9 h/d = 1.00 (0.76–1.32), 5–6.9 h/d = 1.40 (1.00–1.96)^b^, ≥7 h/d = 1.88 (1.17–3.03)^a,b^ 2 h-increment = 1.14 (1.01–1.30)^b^ No history of stroke or MI <3 h/d = 1.00 (ref), 3–4.9 h/d = 0.95 (0.89–1.01), 5–6.9 h/d = 1.06 (0.97–1.16), ≥7 h/d = 1.18 (1.00–1.38)^a,b^ 2 h-increment = 1.04 (1.00–1.08)^b^

Kinoshita et al (2023)	257 adults living in Iwaki region of Hirosaki city, Aomori Aged >20 years at baseline	longitudinal analysis	2 years	Change in obesity-related factors [VFA (cm^2^), BMI (kg/m^2^), adiponectine (µg/mL)]	Device-measured SB Total ST (h/d)	Age, sex, smoking status, education level, alcohol intake, total energy intake, baseline sedentary time baseline VFA, change in VFA for only adiponectine	not applicable	Unstandardized regression coefficient (β) (95% CI) Change in VFA = 3.85 (1.22–6.49)^c^ Change in BMI = 0.09 (−0.05 to 0.23) Change in adiponectine = −0.02 (−0.05 to 0.00)

Chen et al (2023)	1,687 older adults living in town of Sasaguri, Fukuoka w/o needing to any support or care by long-term care insurance system, history of dementia or Parkinson’s disease Aged >65 years at baseline	Cohort	median 8.8 years (12,185 person-years)	Incidence (functional disability certified by long-term care insurance system)	Device-measured SB Total ST (h/d), sedentary bout length (total ST/number of bouts)	Age, sex, education, living alone, BMI, multimorbidity, having a fall in the last y, low walking ability, cognitive impairment, smoking, drinking, MVPA	466 developed functional disability (certified as needing long-term care insurance system)	HR (95% CI) for functional disability: Total ST Q1 = 1.00 (ref), Q2 = 0.94 (0.70–1.27), Q3 = 1.02 (0.76–1.37), Q4 = 0.85 (0.61–1.18) Mean sedentary bout length Q1 = 1.00 (ref), Q2 = 0.95 (0.71–1.25), Q3 = 0.92 (0.70–1.22), Q4 = 0.93 (0.70–1.23) 10–min/d changes in time spent sedentary and in LPA and MVPA Replace LPA = 1.00 (0.99–1.02) Replace MVPA = 0.88 (0.84–0.92)^b^

BMI, body mass index; CAD, coronary artery disease; CI, confidential interval; COPD, chronic obstructive pulmonary disease; CVD, cardiovascular disease; d, day; eGFR, estimated glomerular filtration rate; GDS, Geriatric Depression Scale; h, hour; HR, hazard ratio; IPAQ-SV, Short version of International Physical Activity Questionnaire; IS model, isotempral substitution model; JACC, Japan Collaborative Cohort Study; J-MICC, Japan Multi-Institutional Collaborative Cohort Study; JPHC, Japan Public Health Center-based Prospective Study; LPA, light intensity of physical activity; M, men; MI, myocardial infraction; min, minute; MMSE, mini-mental state examination; MVPA, moderate-to-vigorous intensity of physical activity; OSHPE, Obu Study of Health Promotion for the Elderly; OR, odds ratio; PA, physical activity; PC, personal computer; PT, public transport; Q, quartile; ref, reference; SB, sedentary behavior; ST, sitting/sedentary time; T, total sample; TV, television; VFA, visceral fat area; W, women; w/o, without; WT, walking time.

^a^Significant linear trend (*P* for trend <0.05).

^b^*P* < 0.05, ^c^*P* < 0.01, ^d^*P* < 0.001.

Sedentary behavior was primarily self-reported (in 24 studies); four used accelerometers (Active style Pro HJA-350IT; OMRON Healthcare Co., Ltd. Kyoto, Japan: *n* = 2, HW100: *n* = 2). TV viewing time was the most common self-report measure (*n* = 13), followed by total sitting time (*n* = 8), occupational sitting (*n* = 5), and other sitting behaviors (eg, car riding, PC use). All four device-based studies assessed total sedentary time; one additionally examined prolonged sedentary bouts (≥30 min) and sedentary bout length (total sedentary time divided by the number of bouts per day). In 24 studies, some form of physical activity measure was adjusted for or used in combination to examine associations of sedentary behavior indicators with health outcomes. However, even among articles derived from the same cohort study data, inconsistencies were observed in the naming (eg, some used ‘sport activity,’ while others referred to ‘physical exercise activity’) and the number of variables considered (eg, some adjusted for both walking and sport hours, whereas others included only walking or physical exercise hours). Other than physical activity measures, common covariates included body mass index (BMI), smoking, alcohol use, sex, age, marital status, and educational attainment.

Health outcomes reported were mortality (all-cause, total, colorectal, and liver cancer, coronary artery disease, stroke, CVDs, chronic obstructive pulmonary disease (COPD), and pulmonary embolism) and incidence (total, lung, ovarian, breast endometrial, stomach, colorectal, colon, rectum, pancreas, kidney, bladder, prostate, and other cancer, type 2 diabetes, metabolic syndrome, dementia, functional disability, fall, and depression) and other outcomes (fatigue and obesity-related makers). Given limited studies per outcome, results were summarized narratively.

### Mortality

Six studies examined associations of sedentary behavior with all-cause mortality, with two focusing on self-reported total sitting time and occupational sitting time. A study of 83,034 adults aged 45–74 years (JPHC study) found that ≥8 hours/day of total sitting significantly increased mortality risk compared to <3 hours/day, but only in men.^22^ Another study (J-MICC; *n* = 64,456; 35–69 years) reported a significantly higher risk for ≥7 hours/day compared to <5 hours, with a significant linear trend per 2-hour increment.^23^ Regarding occupational sitting, another analysis of the JPHC study (*n* = 36,516; 50–75 years) found that ≥3 hours/day was associated with increased mortality risk among male primary industry workers.^24^ A 15-year follow-up study (*n* = 1,680; 40–95 years of age) similarly reported a higher mortality risk for increased occupational sitting (measured on a 5-point Likert scale) in the total sample and men.^25^ Of the remaining two studies, a JACC study (*n* = 73,383; 40–79 years) found a higher mortality risk for ≥5 hours/day of TV viewing compared to <3 hours, with a significant linear trend per 2-hour increment.^26^ Another study of 10,233 older adults (5-year follow-up) showed that lower MVPA (<150 min/week) combined with higher total sitting time (≥300 min/day) significantly increased mortality risk compared with the counterpart.^27^ These six findings were largely independent of physical activity indicators, BMI, and other health behaviors (Table 1).

Three studies assessed CVD mortality, with two also reporting on all-cause mortality. Both higher occupational sitting (Likert scale) and ≥7 hours/day of TV viewing were significantly associated with increased CVD mortality risk.^25^^,^^26^ Another analysis of the JACC study found that ≥6 hours/day of TV viewing time significantly increased the risk of coronary artery disease (CAD) and total CVD mortality (reference: <2 hours/day), with a linear trend per 1-hour increment. However, no significant association was observed for stroke after adjusting for BMI, smoking, alcohol consumption, and physical activity indicators.^28^

Two studies from the JACC cohort examined cancer mortality: liver^29^ and colorectal.^30^ TV viewing time was not significantly associated with liver cancer mortality, whereas ≥4.5 hours/day was significantly associated with a higher risk of colorectal and colon cancer mortality (reference: <1.5 hours/day) with a significant linear trend per 1-hour increment. Additionally, a study assessing occupational sitting (Likert scale) in relation to all-cause and cardiovascular mortality reported no significant association with total cancer mortality.^25^ Regarding other causes of mortality, analyses using the JACC cohort found that TV viewing ≥4 hours/day had a significant association with an increased risk of COPD mortality in only men (reference: <2 hours/day),^31^ while TV viewing ≥5 hours/day was associated with a higher risk of pulmonary embolism mortality, also in men (Table 1).^32^

Based on these findings, there is a reasonable level of evidence on longitudinal relationships between sedentary behavior and all-cause mortality. For CVD mortality, sedentary behavior appears to have detrimental effects, though the certainty of evidence remains low. Although large sample sizes (>10,000)^22^^–^^24^^,^^26^^–^^32^ and relatively long follow-up (>10 years)^24^^–^^26^^,^^28^^–^^32^ should be acknowledged as strengths in most studies, utilizing self-reported^22^^–^^32^ or/and surrogated measures of sedentary behavior, such as TV viewing,^26^^,^^28^^–^^32^ limited accurate assessment of the association of sedentary behavior with increased mortality. Further research is warranted to clarify these associations, particularly for outcomes other than all-cause mortality, as the number of studies remain limited, observed associations are only partial, and sedentary behavior measures and cutoff values varies across studies.

### Incidence

Although the reference categories for TV viewing time (eg, <1, 1.5, or 2 hours/day) and classification intervals (eg, every 1, 1.5, or 2 hours) varied across studies, four analyses using JACC study data consistently reported significant associations between TV viewing and increased cancer incidence. Specifically, TV viewing was linked to a higher risk of lung cancer in men (without adjustment for physical activity),^33^ ovarian cancer,^34^ and postmenopausal breast cancer,^35^ whereas no significant association with endometrial cancer.^36^ These results were largely independent of BMI, smoking, alcohol drinking, and physical activity indicators. Additionally, an analysis of the JPHC study examined the relationship between occupational sitting and cancer incidence across 11 cancer sites, reporting that ≥7 hours/day of occupational sitting was significantly associated with an increased incident risk of total and pancreatic cancers in men and lung cancer in women, compared to <1 hour/day (Table 1).^37^ These findings from large-scale cohort studies with long-term follow-up^33^^–^^37^ suggest potential adverse associations between sedentary behavior and site-specific cancer incident risk. However, there were no studies using objective measures of sedentary behavior, nor any that investigated its total amount. Furthermore, for each specific cancer site, only one or two studies were available.

Four studies investigated the association with incidence of functional disability in older adults, which was defined as the first-time certification of requiring long-term care or support under Japanese Public Long-Term Care Insurance System.^38^^–^^41^ Among the two studies utilizing objectively measured sedentary behavior,^38^^,^^41^ findings were mixed depending on whether MVPA was adjusted for. A study conducted among 3,961 older adults in Aichi Prefecture reported a positive association between total sedentary time and disability incidence when MVPA was not accounted for.^38^ However, another study in Fukuoka Prefecture (*n* = 1,687) found no significant associations between total sedentary time or sedentary bout length and disability incidence after adjusting for MVPA.^41^ Despite these differences, isotemporal substitution analyses in both studies found that replacing sedentary time into MVPA time was significantly associated with reduced risk of disability incidence.^38^^,^^41^ Similarly, a study of 6,661 older adults in Yamanashi Prefecture found no association between self-reported total sitting time and disability incidence when adjusted for self-reported MVPA.^40^ In this study, combined effects of total sitting time and MVPA on disability incidence were examined with interaction terms, but stratification by sedentary time category did not appear to influence the protective associations of MVPA against disability risk. Another study in Aichi Prefecture (*n* = 5,104) examined the mediating role of sedentary behavior in the association between renal function and disability incidence. Among older adults with excessive self-reported sedentary time (≥8 hours/day), low renal function (eGFR <45 mL/min/1.73 m^2^) was significantly associated with an increased risk of disability.^35^ While these findings, based on three of the four studies with follow-up periods of 2 to 4 years,^38^^–^^40^ suggest that MVPA may mitigate the detrimental effects of sedentary behavior on functional disability, the number of studies identified remains limited. Although half of studies utilized the objective measures of sedentary behavior, which is a methodological strength, the associations of the well-characterized sedentary pattern including prolonged sedentary behavior and sedentary break with incidence of functional disability were not investigated.

In relation to other geriatric health outcomes, a 5-year follow-up study in Yamanashi Prefecture found that spending ≥30 minutes/day on book reading—a mentally active sedentary behavior—was independently associated with a lower risk of dementia onset compared to <10 minutes/day. However, no significant association was found for TV viewing, categorized as a mentally passive sedentary behavior.^42^ In addition, a 1-year follow-up study of 1,890 community-dwelling older adults aged 60 to 79 years in Shimane Prefecture revealed that only the longest category of self-reported total sedentary time (≥420 minutes/day) was independently associated with a higher risk of fall incidence compared with the shortest category (0–119 minutes/day).^43^

Regarding the incidence of cardiometabolic diseases, a 3-year follow-up study of 430 Japanese workers aged 40–64 years examined the associations of device-measured total sedentary time, non-prolonged sedentary time (<30-minute bouts), and prolonged sedentary time (≥30-minute bouts) with metabolic syndrome. The study found that only prolonged sedentary time in the highest quartile to be significantly associated with an increased risk of metabolic syndrome incidence.^44^ Additionally, an analysis using JACC study data reported that TV viewing ≥5 hours/day was significantly associated with a higher risk of type II diabetes compared to <2 hours/day in both the total population and among women.^45^

In relation to mental health outcomes, two studies examined the incidence of depressive symptoms and major depressive episodes.^46^^,^^47^ A 15-month follow-up study of 3,503 older adults found that self-reported longer sitting time (≥240 minutes/day) was significantly associated with a higher incidence of depressive symptoms, though without adjustment for physical activity indicator.^47^ In contrast, a 12-month follow-up study of 231 workers reported no significant association between self-reported occupational sitting time and onset of major depression episodes after adjusting for physical activity (Table 1).^46^

Based on these findings, longer time spent sedentary may have geriatric, metabolic, and mental health implications for adults. Given the limited number of studies for each outcome and the heterogeneity in study populations and measurement methods for sedentary behavior, further research is urgently needed to clarify these associations.

### Other health outcomes

An 18-month online longitudinal study investigating the association between changes in various aspects of fatigue and domain-specific sedentary behavior among 1,086 workers aged 20–59 years found that increased public transportation sitting during workdays, as well as leisure sitting time spent talking with others and reading materials, were significantly associated with an increase in the motivational aspect of fatigue. Additionally, total sitting time during workdays and sitting time at work were significantly associated with an increase in the physical activity-related aspect of fatigue.^48^ Regarding obesity-related indicators, a two-year longitudinal study of 257 adults aged ≥20 years reported that an increase in device-measured total sedentary time significantly predicted adverse changes in visceral fat area and adiponectin levels, although these findings were not adjusted for physical activity measures (Table 1).^49^ Additional high-quality research is needed to elucidate the relationships between sedentary behavior and a range of health outcomes, including not only fatigue and obesity-related outcomes, but also a variety of other health outcomes.

### Quality assessment

A summary of the detailed quality assessment for each study can be found in eTable 4. Among the 28 studies included in this review, seven were rated as “good”,^25^^,^^27^^,^^28^^,^^31^^,^^41^^–^^44^ while all others were rated as “fair”^1^^–^^3^^,^^5^^–^^10^^,^^13^^–^^17^^,^^19^^–^^24^^,^^26^^,^^29^^,^^30^^,^^32^^–^^39^^,^^45^^–^^49^ except for one study rated as “poor”.^40^ A majority did not report the sample size justification and blinding of outcome assessors to the exposure status of participants. Additionally, two-thirds of the studies employed sedentary behavior measures that were not well-defined, valid, nor reliable. Furthermore, 26 studies did not assess sedentary behavior indicators more than once over time, limiting the strength of causal inferences.

## DISCUSSION

This systematic scoping review was conducted to provide insights into the extent to which evidence on relationships between sedentary behavior and health outcomes exists in Japanese adults, with a particular focus on prospective studies. The findings of this review suggest that sedentary behavior may have deleterious effects on multiple health outcomes, including all-cause mortality, CVD mortality, and incidence of certain site-specific cancers in Japanese populations. Notably, these associations appear to be largely independent of engagement in varied types and modes of physical activity (eg, exercise, walking, MVPA, and sports). These findings offer sufficient scientific evidence to support public health recommendations advocating for minimizing sedentary time to enhance health outcomes in Japan. Therefore, consistent with current WHO guidelines, the practical implication is to encourage adults to reduce total sedentary time whenever possible throughout the day.

However, this scoping review identified several important research gaps. For many health outcomes, the number of studies was limited, even for diseases and conditions that are highly prevalent in Japan, such as mortality and incidence of major site-specific and total cancer, CVD, diabetes, and dementia. Moreover, the overall range of health outcomes assessed in relation to sedentary behavior was relatively narrow, suggesting that numerous potentially relevant outcomes remain underexplored. These findings underscore the need for future epidemiological studies to examine the health impacts of sedentary behavior in a more comprehensive and strategic manner. In addition, the present review revealed considerable heterogeneity in methodologies across studies. In particular, most studies relied on self-reported and/or proxy measures of sedentary behavior, such as TV viewing time, which lead to misclassification and inaccurate estimation of sedentary time. Additionally, the lack of trend analyses in many studies and the substantial variation in the cutoff values of sedentary behavior across studies hindered a clear understanding of any potential dose-response relationships and specific thresholds. Moreover, due to the limited availability of data on the interruption patterns of sedentary behavior, it remains difficult to determine specific or optimal patterns for reducing sedentary behavior. These methodological inconsistencies partly explain the relatively small number of high-quality prospective studies in this review. Utilizing objective measures and establishing methodological consistency and standardization in the assessment of sedentary behavior are essential to enhance the comparability and generalizability of findings and to enable more specific and evidence-based recommendations.

While numerous prospective studies and systematic reviews have examined associations of sedentary behavior with health outcomes in Western populations,^6^^–^^8^^,^^10^^,^^50^^–^^52^ relatively few such studies have been conducted in Japan. Furthermore, since the concept of sedentary behavior was first introduced for public health in 2000,^53^ there has been a marked increase in prospective studies reporting its health risks using objective measures (eg, accelerometers and inclinometers) and self-reported questionnaire, particularly after 2010, based on a more consistent definition of sedentary behavior.^6^^–^^8^^,^^51^^,^^52^ In contrast, studies examining TV viewing time, which was widely used as a specific measure of sedentary behavior and accounted for over two-thirds of the total papers in a 2010 review,^50^ have declined in recent years. In the present review, all but one of the included studies were published in 2013 or later. Although some recent studies applied objectively measured or validated self-report questionnaires of total sedentary time, nearly half of the studies in this review utilized TV viewing time as an exposure variable. Additionally, some studies utilized ordinal Likert-scale assessments of sedentary behavior. This methodological heterogeneity is partly attributable to the use of large cohort datasets initiated before 2000, when the independent health risks of sedentary behavior had not been fully recognized. While the use of existing cohort data has played a significant role in advancing the body of evidence in Japan, the use of poorly validated sedentary behavior measures, including surrogate indicators, may have contributed to lower certainty of evidence regarding health risks. A similar issue was observed in physical activity assessments, which are critical confounders in sedentary behavior research. Thus, conducting further large cohort studies with accelerometer and well-validated questionnaire, such as the International Physical Activity Questionnaire and Global physical activity questionnaire, for both sedentary behavior and physical activity assessments is crucial to accumulate the high-quality evidence in Japan.

Regarding the health outcomes examined in relation to sedentary behavior, all-cause mortality was the most frequently reported in Japan. While studies demonstrated a relatively consistent association, the adverse effects were limited to men or to specific occupational subgroups in some studies. Internationally, several systematic reviews and meta-analyses of prospective cohort studies have shown that spending less time in sedentary behavior is associated with substantially reduced risk of mortality in adults with evidence of non-linear dose-response relations.^6^^,^^8^^,^^52^ With respect to other health outcomes, the majority of reports focused on cancer incidence and mortality in Japan, while only a limited number of studies investigated cardiovascular disease and type 2 diabetes which have been extensively examined and consistently reported its deleterious effects of sedentary behavior in Western countries.^7^^,^^50^^–^^52^ Moreover, nearly all studies focused on the associations with the site-specific cancer risks, which was insufficient for establishing clear evidence of the associations of sedentary behavior with not only mortality and incidence of cardiovascular diseases and type 2 diabetes but also overall cancer mortality. Given that cancer have been the leading cause of death in Japan for over four decades,^54^ further research is warranted to strengthen the evidence for the association of sedentary behavior with cancer. Additionally, expanding research to a broader range of non-communicable diseases and their risk factors is crucial for public health in Japan.

Identifying factors associated with geriatric-relevant health outcomes has been prioritized in Japan which faces a severely declining birthrate and a rapidly-aging population. Against this demographic background, several prospective cohort studies targeting older adults have been initiated since 2010, reporting the association of sedentary behavior with onset of functional disability, which is a key geriatric-related indicator for assessing burden of chronic diseases and long-term care.^55^^,^^56^ Although the findings were from only four studies, the adverse effect of sedentary behavior on functional disability may be mediated by lack of MVPA, suggesting that replacing sedentary behavior with physical activity may be particularly important among Japanese older adults. Considering that few prospective studies have examined the relevant outcome in other Asian and Western counties,^57^ this may be a significant finding that should be validated not only in Japan but also in other counties to enhance the generalization and the robustness of the evidence. Moreover, evidence regarding other geriatric-relevant outcomes, such as cognitive decline or dementia, falls, physical frailty, and depression, remains quite limited in Japan. Apparently, further studies are urgently needed with the aging rate would reach 30% by 2025 in Japan^58^ ahead of many other countries.

### Strengths and limitations

This is the first review to explore the prospective associations between sedentary behavior and health outcomes focusing on Japanese adults. In order to maximize the transparency and robustness, this scoping review followed the PRISMA framework extension for scoping reviews and JBI methodology to employ a robust and transparent protocol.^17^^,^^59^ Accordingly, the present study applied several electronic databases for literature searches and included two independent reviewers for document screening and data verification with a third reviewer resolving any disagreements.

However, there are several limitations. First, no strict quality assessment of the publications included was used, since our purpose was to provide a comprehensive overview of the associations of sedentary behavior and health outcomes in Japan unconstrained by design and quality of the studies, so as to better identify research gaps. Moreover, the level of evidence for each health outcome was not systematically evaluated but was instead assessed narratively through consensus among three of the authors. Thus, the generalizability of the results from this review should be considered with caution. Finally, this review focused exclusively on apparently healthy Japanese adults. Therefore, the results may not be applicable to special populations, such as pregnant individuals, those with disabilities, or clinical populations.

### Conclusions

In conclusion, this systematic scoping review revealed that longer time spent sedentary had deleterious associations with most of the health outcomes examined in large studies from the general Japanese adult population. Furthermore, the associations were largely independent of certain measures of physical activity. The present findings provide sufficient scientific evidence suggesting the need to minimize sedentary time as much as possible to enhance or maintain health. However, definitive conclusions for the association with specific health outcomes and dose-response relations cannot be drawn due to the inherent limitations due to including a relatively small volume of studies and the moderate quality of the studies included in the review. To better understand the health hazards of sedentary behavior in the Japanese adult population, there is a particular need to examine the health risks of sedentary behavior for a wide range of mortalities and morbidities, and focusing on highly prevalent diseases and disabilities in Japanese adults. Studies are needed using objective measures and well-validated and reliable questionnaires of sedentary behavior and physical activity, analyzing dose-response relationships, and accounting for appropriate confounders.

## SUPPLEMENTARY MATERIAL

eTable 1. Search terms and syntax

eTable 2. Search terms and syntax (supplementary search in Japanese databases)

eTable 3. List of excluded full-text articles and reasons

eTable 4. A summary of the detailed quality assessment
