# Supplementary material for: Sedentary Behavior and Health Consequences: A Systematic Scoping Review of Prospective and Longitudinal Studies in Japan
Source: J Epidemiol. 2026 Jan 5;36(1):1–19. doi: 10.2188/jea.JE20250140 (PMC12698325; doi:10.2188/jea.JE20250140)
Supplement: Supplementary file 1 [file je-36-001-s001.pdf]

eTable 1. Search terms and syntax

1		sedentary OR sitting OR television OR TV OR screen time
2	AND	cohort OR longitudinal OR prospective* OR retrospective*
3	AND	Japan*
4	NOT	child* OR youth OR adolescen*

Limited: English

Pubmed	Search: ((cohort OR longitudinal OR prospective* OR retrospective*) AND (Japan*) AND (sedentary OR sitting OR television OR TV OR screen time)) NOT (child* OR youth OR adolescen*) Filters: English, from 2000/1/1 - 2023/12/31<br><br>((("cohort"[All Fields] OR "cohort s"[All Fields] OR "cohorte"[All Fields] OR "cohorts"[All Fields] OR ("longitudinal"[All Fields] OR "longitudinally"[All Fields]) OR "prospective*"[All Fields] OR "retrospective*"[All Fields]) AND "japan*"[All Fields] AND ("sedentaries"[All Fields] OR "sedentariness"[All Fields] OR "sedentary"[All Fields] OR ("sitting position"[MeSH Terms] OR ("sitting"[All Fields] AND "position"[All Fields]) OR "sitting position"[All Fields] OR "sitting"[All Fields] OR "sittings"[All Fields]) OR ("televised"[All Fields] OR "televising"[All Fields] OR "television"[MeSH Terms] OR "television"[All Fields] OR "televisions"[All Fields] OR "television s"[All Fields]) OR "TV"[All Fields] OR ("screen time"[MeSH Terms] OR ("screen"[All Fields] AND "time"[All Fields]) OR "screen time"[All Fields]))) NOT ("child*"[All Fields] OR ("adolescent"[MeSH Terms] OR "adolescent"[All Fields] OR "youth"[All Fields] OR "youths"[All Fields] OR "youth s"[All Fields]) OR "adolescen*"[All Fields])) AND ((2000/1/1:2023/12/31[pdat]) AND (english[Filter]))<br>Filters: Humans Sort by: Most Recent
Web of Science	(((ALL=(sedentary OR sitting OR television OR TV OR "screen time")) AND ALL=(cohort OR longitudinal OR prospective* OR retrospective*)) AND ALL=(Japan*)) NOT ALL=(child* OR youth OR adolescen*)<br>Refine: Date(2000-01-01~2023-1231), Language(English), Document types (Articles)
CINAHL	(sedentary OR sitting OR television OR TV OR screen time) AND (cohort OR longitudinal OR prospective* OR retrospective*) AND Japan* NOT (child* OR youth OR adolescen*)<br>Limited - Publication date: 20000101-20231231; Publication type: Journal Article; Language: English
MEDLINE(Ovid)	(sedentary OR sitting OR television OR TV OR screen time) AND (cohort OR longitudinal OR prospective* OR retrospective*) AND (Japan*) NOT (child* OR youth OR adolescen*)<br>2 limit 1 to (english language and humans and yr="2000 - 2023")

eTable 2. Search terms and syntax (supplementary search in Japanese databases)

1		座位行動 OR 座っている OR 座りがち OR テレビ視聴 OR スクリーンタイム OR スクリーン時間
2	AND	縦断研究 OR コホート研究 OR 前向き研究 OR 後ろ向き研究 OR 追跡研究
3	AND	日本人 OR 日本 OR 日本国内
4	NOT	小児 OR 児童 OR 青少年 OR 思春期 OR 子ども

Ichushi	((((((((身体活動量の少ない生活/TH or 座位行動/AL) or 座っている/AL or 座りがち/AL or テレビ視聴/AL or (画面を見ている時間/TH or スクリーンタイム/AL) or スクリーン時間/AL)) and (DT=2000:2023 and LA=日本語 and (CK=ヒト) and (CK=成人(19～44),中年(45～64),高齢者(65～)))) and (((縦断研究/TH or 縦断研究/AL) or (コホート研究/TH or コホート研究/AL) or (前向き研究/TH or 前向き研究/AL) or (後向き研究/TH or 後ろ向き研究/AL) or (追跡研究/TH or 追跡研究/AL)))) and ((日本人/AL or (日本/TH or 日本/AL) or 日本国内/AL))))) not ((小児/TH or 小児/AL) or (小児/TH or 児童/AL) or (青年/TH or 青少年/AL) or (思春期/TH or 思春期/AL) or (小児/TH or 子ども/AL))
CiNii Research	検索式：(全て日本語、フリーワード検索)<br>(座位行動 OR 座っている OR 座りがち OR テレビ視聴 OR スクリーンタイム OR スクリーン時間) AND (縦断研究 OR コホート研究 OR 前向き研究 OR 後ろ向き研究 OR 追跡研究) AND （日本人 OR 日本 OR 日本国内)<br>検索期間：2000 年 1 月 1 日～2023 年 12 月 31 日

eTable 3. List of excluded full-text articles and reasons

Excluded full-text articles		Reason
1. Adachi T, Kamiya K, Takagi D, Ashikawa H, Hori M, Kondo T, et al. Combined effects of obesity and objectively-measured daily physical activity on the risk of hypertension in middle-aged Japanese men: A 4-year prospective cohort study. Obesity Research & Clinical Practice. 2019;13(4):365-70.		No sedentary behavior outcome
2. Altenburg TM, Lakerveld J, Bot SD, Nijpels G, Chinapaw MJ. The prospective relationship between sedentary time and cardiometabolic health in adults at increased cardiometabolic risk - the Hoorn Prevention Study. International Journal of Behavioral Nutrition & Physical Activity.11:90.		No Japanese participants
3. Amagasa S, Inoue S, Murayama H, Fujiwara T, Kikuchi H, Fukushima N, et al. Changes in rural older adults' sedentary and physically-active behaviors between a non-snowfall and a snowfall season: compositional analysis from the NEIGE study. BMC Public Health. 2020;20(1):1248.		No prospective design
4. Amatori S, Sisti D, Perroni F, Brandi G, Rocchi MBL, Gobbi E. Physical activity, sedentary behaviour and screen time among youths with Down syndrome during the COVID-19 pandemic. Journal of Intellectual Disability Research.66(12):903-12.		No prospective design
5. Annear M, Kidokoro T, Shimizu Y. Walking and Sitting Time among Urban-Living Middle-Aged and Older Japanese. International Journal of Gerontology. 2021;15(1):84-6.		No prospective design
6. Anuradha S, Healy GN, Dunstan DW, Tai ES, Van Dam RM, Lee J, et al. Associations of physical activity and television viewing time with retinal vascular caliber in a multiethnic Asian population. Investigative Ophthalmology & Visual Science.52(9):6522-8.		No Japanese participants
7. Balboa-Castillo T, Leon-Munoz LM, Graciani A, Rodriguez-Artalejo F, Guallar-Castillon P. Longitudinal association of physical activity and sedentary behavior during leisure time with health-related quality of life in community-dwelling older adults. Health & Quality of Life Outcomes.9:47.		No Japanese participants
8. Bao W, Tobias DK, Bowers K, Chavarro J, Vaag A, Grunnet LG, et al. Physical activity and sedentary behaviors associated with risk of progression from gestational diabetes mellitus to type 2 diabetes mellitus: a prospective cohort study. JAMA Internal Medicine.174(7):1047-55.		No Japanese participants
9. Cuthbertson CC, Tan X, Heiss G, Kucharska-Newton A, Nichols HB, Kubota Y, et al. Associations of Leisure-Time Physical Activity and Television Viewing With Life Expectancy Free of Nonfatal Cardiovascular Disease: The ARIC Study. J Am Heart Assoc. 2019;8(18):e012657.		No Japanese participants
10. Fukai K, Harada S, Iida M, Kurihara A, Takeuchi A, Kuwabara K, et al. Metabolic Profiling of Total Physical Activity and Sedentary Behavior in Community-Dwelling Men. PLoS One. 2016;11(10):e0164877.		No prospective design
11. Fujii Y, Yamamoto R, Shinzawa M, Kimura Y, Aoki K, Tomi R, et al. Occupational sedentary behavior and prediction of proteinuria in young to middle-aged adults: a retrospective cohort study. J Nephrol. 2021;34(3):719-28		Clinical setting
12. Gabriel KP, Karvonen-Gutierrez CA, Colvin AB, Ylitalo KR, Whitaker KM, Lange-Maia BS, et al. Associations of accelerometer-determined sedentary behavior and physical activity with physical performance outcomes by race/ethnicity in older women. Preventive Medicine Reports. 2021;23.		No Japanese participants

13. Hamer M, Stamatakis E. Prospective study of sedentary behavior, risk of depression, and cognitive impairment. Medicine & Science in Sports & Exercise.46(4):718-23.		No Japanese participants
14. Hamer M, Yates T, Demakakos P. Television viewing and risk of mortality: Exploring the biological plausibility. Atherosclerosis.263:151-5.		No Japanese participants
15. Hanyuda A, Sawada N, Uchino M, Kawashima M, Yuki K, Tsubota K, et al. Physical inactivity, prolonged sedentary behaviors, and use of visual display terminals as potential risk factors for dry eye disease: JPHC-NEXT study. Ocul Surf. 2020;18(1):56-63.		No prospective design
16. Hishii S, Miyatake N, Nishi H, Katayama A, Ujike K, Koumoto K, et al. Relationship between Sedentary Behavior and All-cause Mortality in Japanese Chronic Hemodialysis Patients: A Prospective Cohort Study. Acta Med Okayama. 2019;73(5):419-25.		Clinical setting
17. Keadle SK, Arem H, Moore SC, Sampson JN, Matthews CE. Impact of changes in television viewing time and physical activity on longevity: a prospective cohort study. International Journal of Behavioral Nutrition & Physical Activity.12:156.		No Japanese participants
18. Kenkhuis MF, EH VANR, Breedveld-Peters JJL, Breukink SO, Janssen-Heijnen MLG, Keulen ETP, et al. Longitudinal Associations of Sedentary Behavior and Physical Activity with Quality of Life in Colorectal Cancer Survivors. Medicine & Science in Sports & Exercise.53(11):2298-308.		No Japanese participants
19. Kim Y, Wilkens LR, Park SY, Goodman MT, Monroe KR, Kolonel LN. Association between various sedentary behaviours and all-cause, cardiovascular disease and cancer mortality: the Multiethnic Cohort Study. Int J Epidemiol. 2013;42(4):1040-56.		No Japanese participants
20. Mamun AA, O'Callaghan MJ, Williams G, Najman JM. Television watching from adolescence to adulthood and its association with BMI, waist circumference, waist-to-hip ratio and obesity: a longitudinal study. Public Health Nutrition.16(1):54-64.		No Japanese participants
21. Martinez-Gomez D, Guallar-Castillon P, Rodriguez-Artalejo F. Sitting Time and Mortality in Older Adults With Disability: A National Cohort Study. Journal of the American Medical Directors Association.17(10):960.e15-20.		No Japanese participants
22. McVeigh J, Smith A, Howie E, Straker L. Trajectories of Television Watching from Childhood to Early Adulthood and Their Association with Body Composition and Mental Health Outcomes in Young Adults. PLoS ONE [Electronic Resource].11(4):e0152879.		No Japanese participants
23. Nagata S, Adachi HM, Hanibuchi T, Amagasa S, Inoue S, Nakaya T. Relationships among changes in walking and sedentary behaviors, individual attributes, changes in work situation, and anxiety during the COVID-19 pandemic in Japan. Preventive Medicine Reports. 2021;24.		No prospective design
24. Nakanishi S, Shimoda M, Tatsumi F, Kohara K, Obata A, Katakura Y, et al. Effects of sedentary behavior and daily walking steps on body mass index and body composition: Prospective observational study using outpatient clinical data of Japanese patients with type 2 diabetes. J Diabetes Investig. 2021;12(9):1732-8.		Clinical setting
25. Namio K, Kondo T, Miyatake N, Hishii S, Nishi H, Katayama A, et al. Prolonged Sedentary Bouts Are Critically Involved in All-Cause Mortality in Patients on Chronic Hemodialysis: A Prospective Cohort Study. Acta Medica Okayama. 2023;77(2):139-45.		Clinical setting
26. Oka T, Ono R, Tsuboi Y, Wada O, Kaga T, Tamura Y, et al. Effect of preoperative sedentary behavior on clinical recovery after total knee arthroplasty: a prospective cohort study. Clin Rheumatol. 2020;39(3):891-8.		Clinical setting
27. Sagelv EH, Hopstock LA, Morseth B, Hansen BH, Steene-Johannessen J, Johansson J, et al. Device-measured physical activity, sedentary time, and risk of all-cause mortality: an individual participant data analysis of four		No Japanese participants

	prospective cohort studies. British Journal of Sports Medicine.57(22):1457-63.		
28.	Shiraishi N, Suzuki Y, Kuromatsu I, Komiya H, Kuzuya M. Sedentary behavior is associated with arteriosclerosis in frail older adults. Nagoya Journal of Medical Science. 2022;84(1):91-100.		No prospective design
29.	Tamiya H, Tamura Y, Mochi S, Akazawa Y, Mochi Y, Banba N, et al. Extended Sedentary Time Increases the Risk of All-Cause Death and New Cardiovascular Events in Patients With Diabetic Kidney Disease. Circulation Journal. 2020;84(12):2190-7.		Clinical setting
30.	Yanagisawa T, Tatematsu N, Horiuchi M, Migitaka S, Yasuda S, Itatsu K, et al. Prolonged preoperative sedentary time is a risk factor for postoperative ileus in patients with colorectal cancer: a propensity score-matched retrospective study. Support Care Cancer. 2023;32(1):54.		Clinical setting
31.	Yanagisawa T, Sugiura H, Tatematsu N, Horiuchi M, Migitaka S, Itatsu K. Preoperative Sedentary Time Predicts Postoperative Complications in Gastrointestinal Cancer. Asian Pac J Cancer Prev. 2020;21(11):3405-11.		Clinical setting
32.	谷口善昭, 牧迫飛雄馬, 中井雄貴, 富岡一俊, 窪薗琢郎, 竹中俊宏, 大石充． 地域在住高齢者における骨量および筋量の低下と身体活動との関連性 理学療法学. 2022;49(2):131-138.		No prospective design

eTable 4. A summary of the detailed quality assessment

	1) Was the research question or objective in this paper clearly stated?	2) Was the study population clearly specified and defined?	3) Was the participation rate of eligible persons at least 50%?	4) Were all the subjects selected or recruited from the same or similar populations (including the same time period)? Were inclusion and exclusion criteria for being in the study prespecified and applied uniformly to all participants?	5) Was a sample size justification, power description, or variance and effect estimates provided?	6) For the analyses in this paper, were the exposure(s) of interest measured prior to the outcome(s) being measured?	7) Was the timeframe sufficient so that one could reasonably expect to see an association between exposure and outcome if it existed?	8) For exposures that can vary in amount or level, did the study examine different levels of the exposure as related to the outcome (e.g., categories of exposure, or exposure measured as continuous variable)?	9) Were the exposure measures (independent variables) clearly defined, valid, reliable, and implemented consistently across all study participants?	10) Was the exposure(s) assessed more than once over time?	11) Were the outcome measures (dependent variables) clearly defined, valid, reliable, and implemented consistently across all study participants?	12) Were the outcome assessors blinded to the exposure status of participants?	13) Was loss to follow-up after baseline 20% or less?	14) Were key potential confounding variables measured and adjusted statistically for their impact on the relationship between exposure(s) and outcome(s)?	Rate
Inoue et al. (2008)	Y	Y	Y	N	NR	Y	Y	Y	N	N	Y	NR	Y	Y	fair
Ukawa et al.(2013)	Y	Y	CD	Y	NR	Y	Y	Y	N	N	Y	NR	Y	N	fair
Ukawa et al.(2014)	Y	Y	Y	Y	NR	Y	Y	Y	N	N	Y	NR	Y	N	fair
Ukawa et al. (2015)	Y	Y	Y	Y	NR	Y	Y	Y	N	N	Y	NR	Y	Y	good
Ikehara etl al. (2015)	Y	Y	Y	Y	NR	Y	Y	Y	N	N	Y	NR	Y	Y	good
Kikuchi et al.(2015)	Y	N	Y	N	NR	Y	Y	Y	N	N	Y	NR	Y	Y	fair
Shirakawa et al. (2016)	Y	Y	NR	Y	NR	Y	Y	Y	N	N	Y	NR	Y	Y	fair
Honda et al. (2016)	Y	Y	Y	Y	NR	Y	CD	Y	Y	N	Y	NR	Y	Y	good
Kitayuguchi et al. (2016)[a]	Y	Y	Y	Y	NR	Y	CD	Y	Y	N	Y	NR	Y	Y	good
Tsutsumimoto et al. (2017)	Y	Y	N	Y	NR	Y	CD	Y	Y	N	Y	NR	Y	Y	fair
Ukawa et al. (2018)	Y	Y	CD	Y	NR	Y	Y	Y	N	N	Y	NR	NR	Y	fair
Ikehara et al.(2019)	Y	Y	CD	Y	NR	Y	Y	Y	N	N	Y	NR	NR	Y	fair
Lee et al., (2019)	Y	NR	Y	Y	NR	Y	Y	NA	Y	Y	NR	NR	Y	Y	fair
Cao et al. (2019)	Y	Y	NR	Y	NR	Y	Y	Y	N	N	Y	NR	NR	Y	fair
Ihara et al.（2020）	Y	N	Y	Y	NR	Y	Y	Y	N	N	Y	NR	Y	Y	fair
Sakaue et al. (2020)	Y	Y	Y	Y	NR	Y	Y	Y	N	N	Y	NR	Y	Y	good
Miyata et al. (2021)	Y	Y	CD	Y	NR	Y	Y	Y	N	N	Y	NR	NR	Y	fair
Koohsari et al. (2021)	Y	Y	N	Y	NR	Y	CD	Y	Y	N	Y	Y	N	Y	fair
Li eta al. (2021)	Y	Y	NR	Y	NR	Y	Y	Y	N	N	Y	NR	Y	Y	fair
Koyama et al. (2021)	Y	Y	Y	CD	Y	Y	CD	Y	Y	N	Y	NR	NR	Y	fair
Watanabe & Kawakami (2021)	Y	Y	N	Y	NR	Y	N	Y	Y	CD	Y	NR	N	Y	fair
Chen et al. (2023)	Y	Y	Y	Y	NR	Y	Y	Y	Y	N	Y	NR	Y	Y	good
Nemoto et al. (2022)	Y	Y	Y	Y	Y	Y	Y	Y	N	N	Y	NR	Y	Y	good
Sato et al. (2022)	Y	Y	Y	Y	NR	Y	CD	N	N	N	Y	NR	Y	Y	poor
Watanabe et al. (2022)	Y	Y	Y	Y	NR	Y	Y	Y	N	N	Y	NR	Y	Y	good
Chiba et al. (2022)	Y	Y	CD	Y	NR	Y	CD	Y	Y	N	Y	NR	Y	Y	fair
Teramoto et al. (2023)	Y	N	Y	Y	NR	Y	Y	Y	N	N	Y	NR	Y	Y	fair
Kinoshita et al. (2023)	Y	Y	N	Y	NR	CD	CD	Y	Y	Y	Y	NR	NA	Y	fair
Chen et al. (2023)	Y	Y	Y	Y	NR	Y	Y	Y	Y	N	Y	NR	Y	Y	good

CD, cannot determine; N, no; NA, not applicalve; NR, not reported; Y, yes.
